# Validation and refinement of a biomarker panel for frailty assessment and prediction of muscle weakness in older adults

**DOI:** 10.1038/s41514-026-00488-1

**Published:** 2026-09-17

**Authors:** Akiko Yamakawa, Tohru Hosoyama, Marie Takemura, Shumpei Niida, Kouichi Ozaki, Shosuke Satake, Daichi Shigemizu

**Affiliations:** 1 Medical Genome Center, Research Institute, National Center for Geriatrics and Gerontology, Obu, Aichi Japan; 2 Geroscience Research Center, Research Institute, National Center for Geriatrics and Gerontology, Obu, Aichi Japan; 3 Center for Frailty and Locomotive Syndrome, Hospital, National Center for Geriatrics and Gerontology, Obu, Aichi Japan; 4 Research Institute, National Center for Geriatrics and Gerontology, Obu, Aichi Japan; 5 RIKEN Center for Integrative Medical Sciences, Yokohama, Kanagawa Japan; 6 Department of Cardiovascular Medicine, Hiroshima University Graduate School of Biomedical and Health Sciences, Hiroshima, Japan; 7 Department of Aging Research, Nagoya University Graduate School of Medicine, Nagoya, Japan; 8 Department of Geriatric Medicine, Hospital, National Center for Geriatrics and Gerontology, Obu, Aichi Japan; 9 Department of Frailty Research, Center for Gerontology and Social Science, Research Institute, National Center for Geriatrics and Gerontology, Obu, Aichi Japan

**Keywords:** Biomarkers, Diseases, Health care, Medical research, Risk factors

## Abstract

Frailty is a complex geriatric syndrome characterized by age-related declines in physiological function and cognitive reserve. To promote early prevention and intervention, minimally invasive and objective biomarkers that can detect frailty progression are required. We aimed to identify biomarkers associated with frailty progression and to elucidate their relevance to the Japanese version of the Cardiovascular Health Study (J-CHS) criteria, consist of five components (unintentional weight loss, self-reported exhaustion, muscle weakness, slow walking speed, and low physical activity). A total of 168 individuals (61 robust, 25 pre-frail, and 82 frail) enrolled in the NCGG (National Center for Geriatrics and Gerontology) Biobank were analyzed. Clinical information, blood-test data, aging-related factors, and gene-expression data were integrated for the analysis. First, linear regression identified one clinical factor, five aging-related factors, and 251 gene-expression factors associated with frailty. Subsequent logistic regression analyses examining each J-CHS components highlighted six candidate biomarkers. Cross-validation further suggested that three of these biomarkers—SMI, apelin, and GDF15—may represent potential biomarkers. Finally, retrospective and prospective analyses further demonstrated that those biomarkers were predictive of future muscle weakness, yielding a concordance index of 0.70. In conclusion, we validated and refined a biomarker panel consisting of SMI, apelin, and GDF15 that is associated with frailty, particularly muscle weakness (a major J-CHS component). These biomarkers may be useful for frailty assessment. Longitudinal analyses further suggested that they may be associated with the future development of muscle weakness in initially robust older adults, although validation in larger prospective cohorts is warranted.

## Introduction

Frailty is a common geriatric syndrome characterized by age-related declines in physiological function and cognitive reserve across multiple systems^1,2^. Individuals with frailty are at increased risk of adverse outcomes, including falls, accidental injuries, hospitalization, bedriddenness, and mortality^3,4^. The prevalence of frailty rises substantially with age, increasing from approximately 7% in adults aged 65 years and older to nearly 30% in those aged 80 years and older^5^. Although the prevalence of frailty increases with advancing age, previous reports have demonstrated that interventions can prevent its progression^6–8^. Multifactorial interventions, including physical activity and nutritional supplementation, have been reported not only to prevent frailty but also to reverse it in some cases^9,10^. Therefore, low-invasive biomarkers that enable early diagnosis of frailty are urgently required.

Currently, widely used diagnostic criteria for frailty are the frailty phenotype proposed by Fried et al.^11^. In the Cardiovascular Health Study (CHS) frailty phenotype, frailty is defined by the presence of three or more of the following five criteria: unintentional weight loss, self-reported exhaustion, weakness (grip strength), slow walking speed, and low physical activity. Pre-frailty is defined as the presence of one or two of these criteria. In Japan, a modified version of these criteria, namely the Japanese version of the Cardiovascular Health Study (J-CHS), has been developed and is widely applied for frailty assessment in older Japanese adults^12^. The J-CHS criteria assess the same five frailty components as the original Fried phenotype, but use cutoff values adapted for the Japanese population, particularly for grip strength and walking speed.

In parallel with these diagnostic frameworks, numerous potential biomarkers for frailty have been proposed across diverse biological and clinical analyses. Among older Japanese individuals, factors such as years of education^13^, skeletal muscle mass index (SMI, an indicator of muscle mass)^14^, and bone mineral density (BMD)^15^ have been identified as clinical predictors of frailty. Body mass index (BMI) has also been consistently associated with frailty across populations in Europe, America, and Asia, including in Japan^16–18^. In terms of minimally invasive blood-based biomarkers, several hematological and biochemical markers have been reported, including hemoglobin (Hb), albumin, high-density lipoprotein (HLD), and hemoglobin-to-red cell distribution width ratio (Hb/RDW)^19,20^. Furthermore, metabolites associated with antioxidation, muscle or nitrogen metabolism, and amino acids have been highlighted as additional candidate biomarkers for frailty^21^.

In addition to these clinical and biochemical indicators, a core panel of frailty-related biological pathways derived from gene-expression databases—including inflammation, mitochondrial dysfunction and apoptosis, calcium homeostasis, fibrosis, neuromuscular and neuronal integrity, cytoskeletal regulation and hormonal signaling, and other related mechanisms—has attracted increasing attention as a source of biomarker candidates^22^. Given the complex and multifactorial pathophysiology of frailty, proposed biomarkers span diverse systems, including musculoskeletal, hormonal, stem-cell and metabolic markers. To integrate these diversities, a conceptual framework has been proposed to guide the identification of future biomarkers across these systems^23^. In recent years, attempts have been made to determine optimal biomarkers by combining multiple biological and clinical indicators; however, most of these studies have been limited to cross-sectional designs^24^. Our previous work also aimed to identify useful biomarkers for frailty by using clinical data, blood-test data, aging-related-factor data, and gene-expression data, but it likewise relied on a cross-sectional approach^14^.

In this study, we aimed to identify biomarkers associated with frailty through an integrating analysis of clinical data, blood-test data, aging-related-factors, and gene-expression data obtained from robust, pre-frail, and frail older adults. We further investigated the associations between the identified biomarkers and individual components of the J-CHS criteria. In addition, we evaluated the potential utility of these biomarkers for predicting future muscle weakness using longitudinal follow-up data.

## Results

### Characteristics of study participants and their J-CHS criteria

**Fig. 1:**
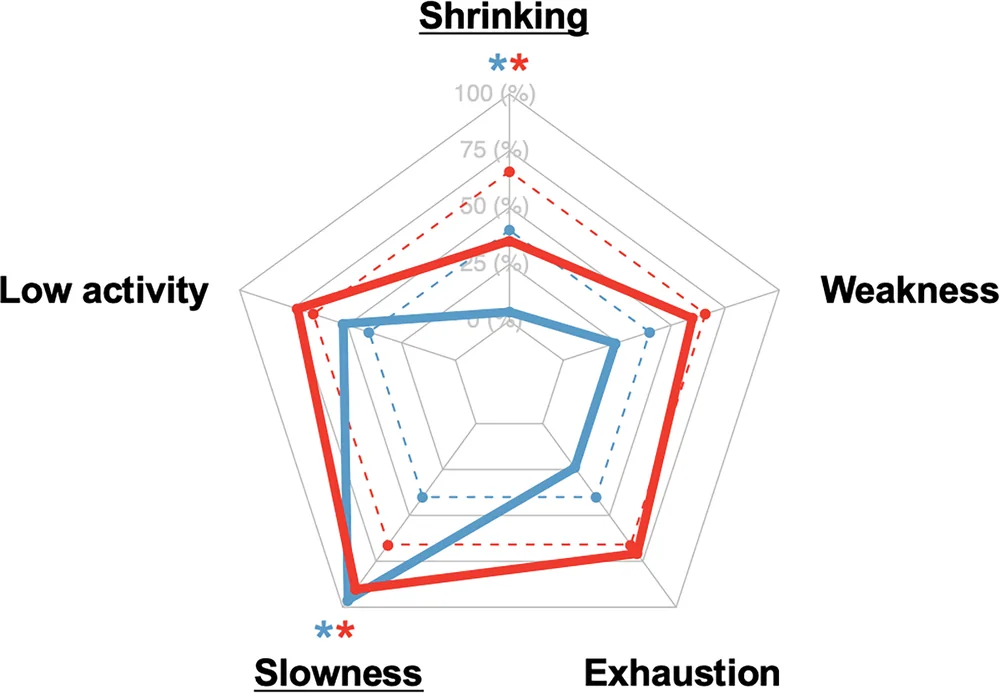
Distribution of J-CHS scores in pre-frail and frail subjects. Proportions of the five J-CHS components—shrinking, weakness, exhaustion, slowness, and low activity—in pre-frail (blue) and frail (red) subjects are shown as observed (solid lines) and expected (dashed lines) values. An asterisk indicates a statistically significant difference between the observed and the expected values for each criterion (*P* < 0.05), with the corresponding J-CHS components underlined.

Of the 168 study participants, 61 were classified as robust, 25 as pre-frail, and 82 as frail. The participant characteristics are summarized in Table 1. We first examined whether there was any bias in the distribution of each J-CHS criterion between the pre-frail and frail groups. Because the scores varied within each category (pre-frail: 1 or 2; frail: 3 to 5), the observed and expected values for each J-CHS criterion were calculated on the basis of the score and the corresponding number of individuals. Based on the distribution of J-CHS scores, the expected value of each J-CHS criterion was estimated to be 40.0% in the pre-frail group and 65.9% in the frail group. The pre-frail group consisted entirely of individuals with a J-CHS score of 2, whereas the frail group comprised 58 individuals with a J-CHS score of 3 and 24 individuals with a score of 4, corresponding a weighted mean J-CHS score of 3.29 (((3 × 58) + (4 × 24))/(58 + 24)). Significant differences between the expected and observed values were observed for shrinking and slowness in both pre-frailty and frailty groups, whereas weakness, exhaustion, and low activity showed no clear association with either group (Fig. 1).

**Table 1:** Summary of characteristics of robust, pre-frail, and frail samples

Category	Total	Robust	Pre-frail	Frail
Number of samples	168	61	25	82
Age (years)^a^	77.3 ± 6.1	76.1 ± 5.7	75.8 ± 7.1	78.6 ± 5.9
Sex ratio (male:female)	1:2.17	1:1.90	1:2.57	1:2.28
BMI (kg/m^2^)^a^	24.49 ± 4.08	24.27 ± 2.73	24.65 ± 3.19	24.6 ± 5.07
J-CHS criteria				
Shrinking	30	0	1	29
Weakness	55	0	6	49
Exhaustion	64	0	6	58
Slowness	98	0	24	74
Low activity	73	0	13	60

^a^Mean ± 1 SD.

### Clinical and blood-test data analysis

To identify clinical factors related to frailty, we examined seven clinical variables and 44 blood-test items that were not included in the frailty diagnosis criteria (Supplementary Table 1 and 2). Linear regression analysis revealed that only SMI was significantly associated with frailty, and none of the blood test items showed significant associations.

**Fig. 2:**
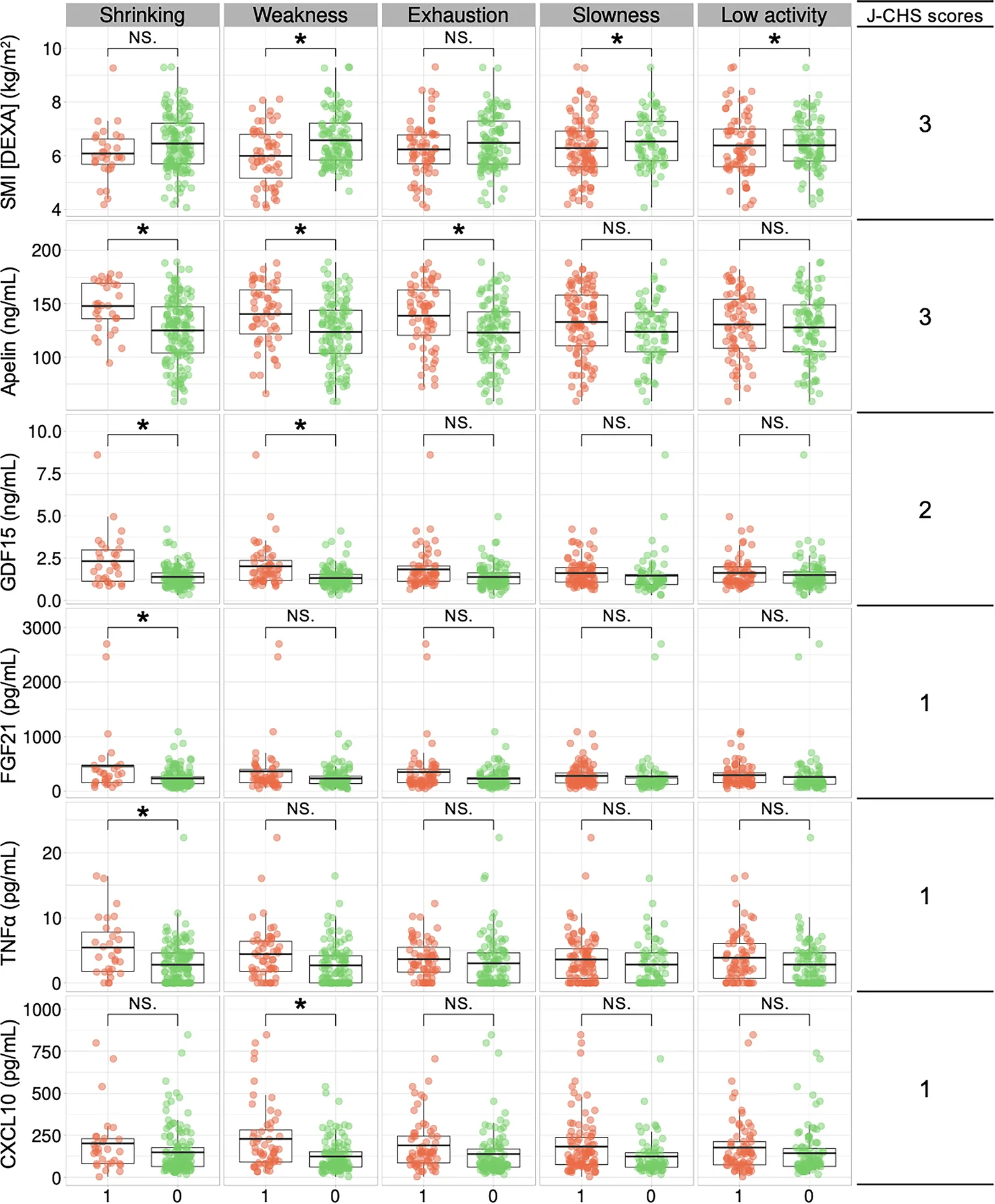
Associations between frailty-related factors and each J-CHS criterion. Box plots show the associations between each J-CHS criterion (1 or 0; *x* axis) and the frailty-related factors (*y* axis), displaying the minimum, lower quartile, mean, upper quartile, and maximum values. Statistical significance was assessed by logistic regression, with *P* < 0.05 considered significant. An asterisk indicates *P* < 0.05; NS indicates not significant. Statistical significance was evaluated for each criterion, and the sum of the frailty-related factors is shown in the right column as the total J-CHS score.

We next evaluated the relationship between SMI and the five J-CHS criteria (shrinking, weakness, exhaustion, slowness, and low activity) by using a logistic regression analysis (Supplementary Table 3). Weakness, slowness, and low activity were significantly associated with SMI (Fig. 2). These results support the role of SMI as an indicator of skeletal muscle status.

### ELISA data analysis

We measured a total of 10 humoral factors previously reported as potential biomarkers of age-related frailty (adiponectin, apelin, BDNF, CXCL9, CXCL10, eNAMPT, FGF21, GDF15, progranulin, and TNFα) in blood serum samples by using ELISA. Linear regression analysis with an additive model across the robust, pre-frail, and frail groups identified five factors—GDF15, apelin, CXCL10, TNFα, and FGF21—that were significantly associated with frailty (Supplementary Table 4).

We next assessed the associations of these five factors with the five J-CHS criteria by using logistic regression analysis (Supplementary Table 3). Apelin showed associations with the greatest number of criteria, namely shrinking, weakness, and exhaustion (Fig. 2). GDF15 was associated with shrinking and weakness; FGF21 and TNFα were each associated with shrinking; and CXCL10 was associated with weakness.

### RNA-seq data analysis

On average, from the 168 study participants we obtained 28.7 million raw read sequences, of which 98.9% were of high quality (i.e., > Q20). After we had removed low-quality reads and trimmed the adaptor sequences, 28.3 million clean reads remained, with 82.9% uniquely mapped to the human reference genome (GRCh37) (Supplementary Table 5).

A total of 6183 genes with transcripts per million (TPM) ≥ 1 across all samples were retained for analysis. Linear regression analysis using an additive model across the robust, pre-frail, and frail groups identified 251 genes significantly associated with frailty (Supplementary Table 6). However, logistic regression analyses evaluating the associations between these genes and the five J-CHS criteria did not identify any statistically significant associations after Bonferroni-correction (*P* < 0.05). Given the relatively small sample size and the large number of genes evaluated, the statistical power to detect robust transcriptomic associations was limited. Therefore, the RNA-seq findings were considered exploratory.

Furthermore, gene set enrichment analysis of the 251 frailty-associated genes using the DAVID gene functional annotation tool (DAVID 2025) did not identify any statistically significant enriched pathways (false discovery rate [FDR] < 0.05). These findings suggest that frailty-related transcriptomic alterations may be distributed across multiple biological processes rather than concentrated within a single pathway, consistent with the heterogeneous and multifactorial nature of frailty.

We also examined the correlation between gene express levels measured by RNA-seq (TPM) and protein concentrations measured by ELISA. Statistically significant correlations were observed for *CXCL10*, *GDF15*, and *NAMPT*, whereas no significant corrections were detected for the other genes (Supplementary Table 7). However, none of these three genes were included among the 251 genes identified as significantly associated with frailty in the RNA-seq analysis. *CXCL10* and *GDF15* were excluded because their expression levels were low across samples (mean TPM < 1), whereas *NAMPT* did not remain significant after correction for multiple-testing.

### Retrospective analysis

**Fig. 3:**
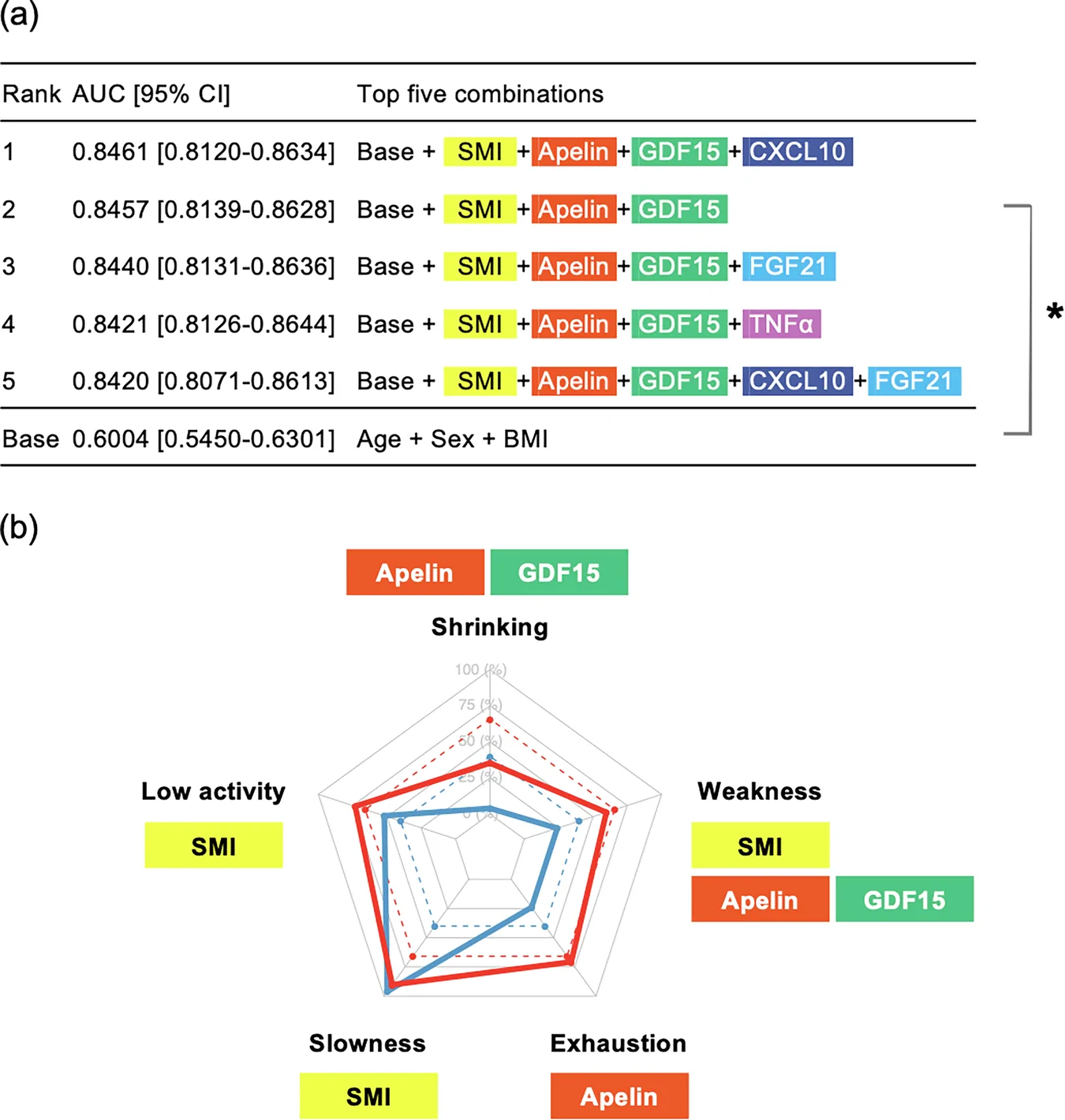
Biomarkers associated with each J-CHS criterion. **a** All possible combinations of the six biomarkers were considered for model construction using five-fold cross-validation. The detailed combinations, along with the mean AUCs and 95% confidence intervals (CIs) of the top five models are shown. “Base” represents the model adjusted only for age, sex, and body mass index (BMI). An asterisk indicates a statistically significant difference between the Rank 2 model and the Base model (*P* < 0.05). **b** One clinical variable (skeletal muscle mass index [SMI]; shown in black bold letters) and two age-related factors (apelin and growth differentiation factor 15 [GDF15]; shown in white bold letters) were associated with the five J-CHS criteria.

A total of six candidate factors for frailty were identified: SMI from the clinical data analysis; and apelin, CXCL10, FGF21, GDF15, and TNFα from the ELISA data analysis. Prediction models distinguishing robust from frail patients were constructed by using all possible combinations of these six candidates (63 combinations in total) with logistic regression. Model performance was evaluated by examining the AUCs from five-fold cross-validation. The top five models achieved AUCs of up to 0.84 (Fig. 3a). Odds ratios for each factor in the top five models and the baseline model are shown in Supplementary Fig. 1.

Notably, all top-performing models included SMI, apelin, and GDF15. Therefore, we selected a model incorporating these three factors, together with age, sex, and BMI, as the optimal model (AUC: 0.8457, 95% CI: 0.8139 to 0.8628). This model showed significantly higher predictive performance than the baseline model (AUC: 0.6004, 95% CI: 0.5450 to 0.6301, Welch’s *t*-test; *P* < 0.05). Importantly, because the J-CHS criteria were associated with these three factors, all five criteria were represented by SMI, apelin, and GDF15 (Fig. 3b).

Given that SMI is biologically related to several J-CHS components, particularly weakness and slowness, we further evaluated its incremental prediction value. A model including SMI in addition to age, sex, and BMI achieved a significantly higher AUC than the baseline model (AUC = 0.762, 95% CI: 0.735–0.782 vs. AUC = 0.600, 95% CI: 0.545–0.630; Welch’s *t*-test; *P* < 0.05). These findings indicate that SMI provides predictive information beyond basic demographic characteristics.

### Prospective analysis

We next investigated whether the three biomarkers identified in the retrospective analysis could be applied to prospective prediction models. Longitudinal follow-up data were available for 37 of the 61 robust participants. Because all robust participants had a baseline score of 0 for each J-CHS component, the number of participants with a score of 1 at follow-up corresponded to the number of incident events. Among the five J-CHS criteria, at least three incident events were observed for shrinking and weakness during follow-up (Supplementary Table 8).

**Fig. 4:**
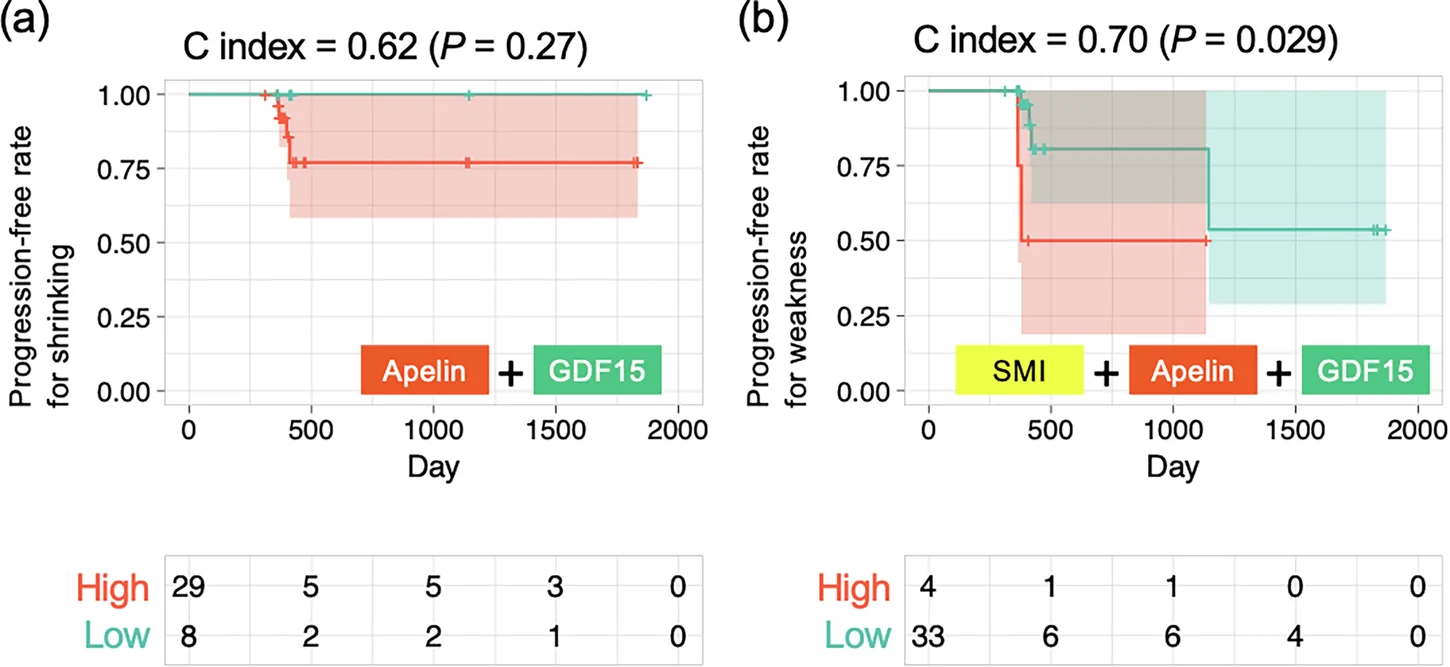
Application of identified biomarkers to prospective data. In the prospective cohort of robust subjects, participants were stratified into high-risk (pink) and low-risk (light blue) groups for shrinking (**a**) and weakness (**b**) on the basis of the identified biomarkers: apelin and growth differentiation factor 15 (GDF15) for shrinking; and skeletal muscle mass index (SMI), apelin, and GDF15 for weakness. The optimal cutoff values were determined by using the minimum *P* value from the log-rank test on Kaplan–Meier curves. No statistically significant differences were observed between the high- and low-risk groups with the combination of apelin and GDF15 for shrinking (**a**), but statistically significant between-group differences were detected with the combination of SMI, apelin, and GDF15 for weakness (**b**).

For shrinking, a Cox proportional hazards model was constructed by using apelin and GDF15, whereas for weakness, the model included SMI, apelin, and GDF15. Kaplan–Meier curves showed no significant difference in the progression-free rate of shrinking between robust groups at high and low risk (log-rank test; *P* = 0.27, Fig. 4a), but there was a significant between-group difference in the case of weakness (log-rank test; *P* = 0.029, Fig. 4b). Moreover, the combination of SMI, apelin, and GDF15 had high predictive performance for weakness (C index = 0.70, Fig. 4b). These findings suggest that apelin and GDF15 could not be used to classify individuals by risk of shrinking, whereas the combination of SMI, apelin, and GDF15 effectively predicted risk of weakness.

## Discussion

In this study, we comprehensively investigated frailty-associated factors by integrating clinical data, blood-test data, aging-related factors, and gene-expression data, and we identified three key biomarkers associated with the J-CHS criteria: SMI, apelin, and GDF15. Because these three markers collectively represented five of the J-CHS criteria, they are likely to be potential biomarkers for the early diagnosis of frailty. Although the lack of longitudinal data limited our ability to validate these biomarkers as predictors of J-CHS criteria other than weakness in the prospective analyses, they contributed to frailty prediction in the retrospective data. Overall, our findings indicate that SMI, apelin, and GDF15 may serve as useful biomarkers for the early detection of at least weakness during the progression from robust to pre-frailty.

A previous study based on retrospective analyses reported that five factors—SMI, apelin, GDF15, adiponectin, and CXCL9—were associated with frailty^14^. Compared with our previous study, the present study extends the evaluation of candidate frailty biomarkers by including pre-frail individuals, examining associations with individual J-CHS components, and performing exploratory prospective analyses using longitudinal data. These additional analyses refined the previously identified five-biomarker panel to three biomarkers (SMI, apelin, and GDF15) that showed the most consistent associations with frailty and frailty-related outcomes. Therefore, the present study should be viewed as a validation and refinement of our previous findings rather than the identification of entirely new biomarkers.

To facilitate earlier and more effective interventions for frailty, we investigated risk factors associated with specific components of the J-CHS criteria using longitudinal data. Among the three biomarkers identified, SMI—which indicates skeletal muscle mass in the limbs, corrected for height—was found to be a valid biomarker for weakness, low activity, and slowness. Weakness has been widely observed in frail older adults^25,26^, and the decline in SMI with advancing frailty in our cohort supports its relevance in capturing these three muscle-related parameters.

GDF15—a cytokine known to increase with age—has been proposed as a biomarker of frailty owing to its negative correlation with muscle strength^27,28^. Consistent with previous studies^29,30^, we found that GDF15 levels increased with frailty progression and were particularly associated with weakness. Furthermore, GDF15 has been implicated in age-related mitochondrial dysfunction, and transgenic mice overexpressing GDF15 exhibit weight loss as a consequence of altered energy metabolism^31^. We also identified GDF15 as a biomarker of weight loss in aging humans, further supporting the relevance and biological consistency of our findings.

Apelin has been shown to be associated with physical exercise^32^, and both apelin synthesis in skeletal muscle and plasma apelin levels have been reported to decrease with age^33^. Additionally, apelin-deficient mice exhibit dramatically accelerated muscle aging^34^, supporting the potential of apelin as a biomarker of muscle weakness. Apelin is involved in several functions, including the reduction of oxidative stress, attenuation of inflammation, regulation of autophagy, and activation of stem cells, and a decline in apelin has been implicated in the acceleration of aging^35^. In addition, dysfunction of the apelin-APJ (the apelin receptor) system contributes to remyelination failure in old animals^36^. Furthermore, vitamin D3 supplementation of mice suppresses the expression of muscle atrophy-related genes associated with cellular senescence in aged skeletal muscle, suggesting that it may mitigate the muscle aging resulting from apelin depletion^37^.

Our focus on the individual J-CHS criteria revealed two or more risk factors for shrinking and weakness, whereas only one factor was identified for each of exhaustion, slowness, and low activity. This may have been due to the fact that exhaustion and low activity are subjective indicators and are therefore difficult to model quantitatively. However, because predicting a disease or condition with a single risk factor is generally challenging, further exploration of risk factors for these three criteria may be necessary. With the accumulation of larger datasets, additional informative biomarkers may be identified from blood-test and gene-expression data.

We also observed that the associations between frailty and the five components of the J-CHS criteria were not uniform. In particular, slowness showed a significant increase, whereas shrinking increased only modestly. These findings suggest that an increase in slowness may be particularly important for the early detection of frailty. However, in the longitudinal data that we used, slowness did not show a statistically significant increase. A previous report found modest annual frailty transition rates (from robust to pre-frail, range 12% to 34%; from pre-frail to frail, range 13% to 15%^38^), indicating that a larger sample and a longer follow-up period may be needed to detect significant changes over time.

A major limitation of our study was the limited availability of longitudinal data, as discussed above. Although long-term follow-up is challenging in older adults, continued data collection will be essential for determining whether SMI, GDF15, and apelin can predict early frailty progression, including weight loss, exhaustion, reduced walking speed, and reduced physical activity. Because the prospective analysis was based on a relatively small number of participants and incident events, the findings should be interpreted with caution and require validation in larger prospective cohorts. Secondly, the cross-sectional dataset used in this study was also relatively small. Although the prediction model incorporating these biomarkers achieved a high AUC, the model was developed and evaluated within the same cohort, which may have resulted in optimistic estimates of predictive performance. Therefore, validation in larger and independent populations is necessary to confirm the robustness, generalizability, and clinical utility of the identified biomarkers. Thirdly, SMI represents skeletal muscle mass and is related to several components of the J-CHS criteria, particularly weakness, slowness, and low-activity. Therefore, the association between SMI and frailty-related outcomes may partly reflect overlap with the frailty diagnostic criteria themselves rather than an entirely independent biomarker effect. This potential overlap should be considered when interpreting the predictive value of SMI. In addition, although we measured SMI by using DEXA, this method is available only at a limited number of facilities. In the future, data collection may be facilitated by adopting simpler and more widely accessible assessment methods, such as the InBody device. Despite these limitations, SMI, GDF15, and apelin were consistently associated with frailty and muscle weakness in our analyses. Further validation in large-scale and independent cohorts will be necessary to establish their clinical utility as biomarkers for frailty assessment and early detection.

## Methods

### Clinical samples

The Locomotor Frailty Sarcopenia Registry was established to accumulate evidence on locomotive syndrome, frailty, and sarcopenia. The registry is organized by the Center for Frailty and Locomotive Syndrome of the National Center for Geriatrics and Gerontology (NCGG). Participants were enrolled in the NCGG biobank, where their blood samples were stored, and their clinical data were obtained from the Center for Frailty and Locomotive Syndrome of the NCGG. The study protocol was created in accordance with the Declaration of Helsinki and approved by the Ethics Committee of Human Research, established at the NCGG (approval numbers 1618 and 1564-2). All participants were volunteers and provided written informed consent before registering in the NCGG Biobank.

Frailty status was diagnosed by using the J-CHS criteria^12^, which consist of the following five components: 1. Shrinking—unintentional weight loss of ≥2 kg in the past 6 months (“Yes” = 1 point); 2. Exhaustion—feeling tired without a reason in the past 2 weeks (“Yes” = 1 point); 3. Low activity—engagement in moderate or low levels of physical exercise or sports aimed at health at least once a week (“No” = 1 point); 4. Slowness—gait speed <1.0 m/s (“Yes” = 1 point); and 5. Weakness—grip strength <28 kg in males or <18 kg in females (“Yes” = 1 point). Participants were categorized on the basis of their total J-CHS scores as robust (score = 0), pre-frail (score = 1 or 2), or frail (score ≥3).

We used 168 high-quality RNA samples (RNA integrity number [RIN] ≥6) from participants aged ≥60 years. Of these participants, 61 were classified as robust, 25 as pre-frail, and 82 as frail. None of the participants had diseases associated with motor symptoms, severe renal failure, severe liver failure, autoimmune diseases, steroid use, or cancer. Some of the frail and robust samples included in the present study were included in our previous study^14^.

To estimate the expected value of each J-CHS criterion within the pre-frail and frail groups, we calculated the statistical expectation based on the distribution of J-CHS scores. Because of the J-CHS score ranges from 0 to 5 and represents the total number of positive frailty criteria, the expected value of an individual criterion was estimated as the mean J-CHS score divided by five and expressed as a percentage. For groups containing multiple J-CHS score categories, the mean J-CHS score was calculated as a weighted average according to the proportion of participants in each score category.

### Clinical and blood-test data

Clinical data consisted of seven variables: six physical performances—SMI, systolic blood pressure (sBP), diastolic blood pressure (dBP), heart rate, young adult mean (YAM) of the lumbar spine, and YAM of the femoral neck—and years of education. SMI was measured by using dual-energy X-ray absorptiometry (DEXA). Blood pressure was measured twice with a sphygmomanometer after the participants had been seated and rested for 5 min, and the lower of two measurements each of sBP and of dBP was used for analysis. Heart rate was obtained at the same time as blood pressure. YAM values were measured at the lumbar spine (L2 to L4) and the femoral neck by DEXA.

Blood-test data comprised 44 items obtained from routine blood tests. All items were measured in at least 156 participants (Supplementary Table 1).

### Enzyme-linked immunosorbent assay

A total of 10 humoral factors previously reported as possible biomarkers of age-related frailty were measured by using enzyme-linked immunosorbent assay (ELISA)^14,22^. These were: total adiponectin (R&D Systems, Minneapolis, MN), apelin-12 (Phoenix Pharmaceuticals, Burlingame, CA), brain-derived neutrotrophic factor (BDNF) (Fujifilm-Wako, Osaka), C-X-C motif chemokine ligand 9 (CXCL9) (R&D Systems), C-X-C motif chemokine ligand 10 (CXCL10) (R&D Systems), Nampt (AdipoGen Life Science, San Diego, CA) for extracellular nicotinamide phosphoribosyltransferase (eNampt), fibroblast growth factor 21 (FGF21) (R&D Systems), growth differentiation factor 15 (GDF15) (R&D Systems), progranulin (AdipoGen Life Sciences), and tumor necrosis factor alpha (TNFα) (R&D Systems). Assays were performed in accordance with the manufacture’s protocols.

Whole blood samples were obtained from the NCGG biobank. Serum blood samples were prepared by centrifugation at 3000 rpm for 10 min, and the supernatants were collected for analysis. All assays were performed in duplicate, and the average value of each measurement was used for statistical analysis. If necessary, samples were diluted with the appropriate buffer provided in each ELISA kit.

### RNA sequencing

Isolation of the buffy coat from whole blood and extraction of total RNAs from the buffy coat were conducted in accordance with the standard operating procedure of the NCGG Biobank^39^. Only high-quality RNA samples with an RNA integrity number (RIN) ≥ 6.0 were selected for sequencing library construction. For each sample, 500 ng of total RNA was used to prepare sequencing libraries by using Illumina TruSeq Stranded Total RNA with Ribo-Zero Globin and IDT for Illumina TruSeq UD Indexes (Illumina, San Diego, CA) in accordance with the manufacturer’s instructions. The prepared libraries were sequenced on the Illumina NovaSeq6000 platform with paired-end reads of 151 bp, in accordance with the manufacturer’s instructions.

### RNA sequencing data analysis

All RNA sequencing (RNA-seq) data were downloaded from the NCGG Biobank database^39^. Sequence quality of the raw reads (FASTQ files) was evaluated by using FastQC (version 0.11.7). Low-quality reads (<Q20) and trimmed reads with adaptor sequences shorter than 50 bp were removed by using Cutadapt (version 1.16). High-quality reads were then mapped to the human reference genome (GRCh37) by using STAR^40^ (version 2.5.2b) with the two-pass mapping option. Gene-level read counts were quantified by using the featureCounts program^41^ from the Subread package (version 1.6.6). Gene expression levels were normalized as transcripts per million (TPM).

### Detection of frailty-related factors

Frailty-related factors were identified from four data sets, namely clinical, blood-test, ELISA, and RNA-seq data. For the RNA-seq data, frailty-associated genes were identified across robust, pre-frail, and frail subjects using linear regression analysis of TPM values, with adjustments for age, sex, and BMI. Multiple-testing correction was performed using the Bonferroni method, and genes with a Bonferroni-corrected *P* value < 0.05 were considered statistically significant. Similarly, linear regression analyses were performed for the clinical, blood-test, and ELISA datasets, with adjustments for age, sex, and BMI. Variables with a Bonferroni-corrected *P* < 0.05 within each dataset were defined as frailty-related factors.

Frailty-related factors identified in these analyses were subsequently evaluated for their associations with frailty status, as defined by J-CHS criteria, using logistic regression analysis. A *P* value < 0.05 was considered statistically significant. All linear regression analyses were performed using the stats package (version 3.4.3) in R.

### Retrospective analysis

Logistic regression analyses were performed by using all possible combinations of frailty-related factors, along with age, sex, and BMI as explanatory variables. The binary outcome variables were frailty status, categorized as robustness or frailty on the basis of the J-CHS criteria. For each model, predictive performance was evaluated by using the area under the receiver operating characteristic curve (AUC) values, calculating from five-fold cross-validation. To assess the robustness and variability of the AUC estimates, the cross-validation procedure was performed 1000 times by using bootstrap resampling. The statistical significance of the performance differences between the best-performing model and a baseline model (including only age, sex, and BMI) was evaluated with Welch’s *t*-test. All analyses were performed with R statistical software, with logistic regression implemented in the stats package (version 3.4.3) and AUC estimation conducted using the ROCR package (version 1.0-7).

### Prospective analysis

Among participants initially classified as robust (i.e., those who scored 0 for each J-CHS component), only those with longitudinal follow-up data were included in the prospective analysis. Of the five J-CHS components, those for which at least three participants experienced a change in status during follow-up were included in the analysis. To evaluate whether the frailty-related factors identified in the retrospective analysis were associated with future frailty-related outcomes, Cox proportional hazards models were applied separately in each J-CHS component. Risk scores were calculated for each participant as a weighted sum of age, sex, BMI, and the identified biomarkers using the regression coefficients estimated from the Cox model. Participants were ranked according to their risk scores and classified into low- and high-risk groups using a cutoff value. The optimal cutoff value was determined through an exploratory procedure as the value yielding the minimum *P* value in log-rank tests comparing the survival distributions of the two risk groups. Because the cutoff selection and performance evaluation were conducted within the same dataset, the results should be interpreted as exploratory. All analyses were implemented using the R statistical software packages survival (version 3.4-3) and survminer (version 3.1.12).

## Supplementary information

Supplementary information

## Data Availability

All datasets used and/or analyzed during the current study are not publicly available due to ethical and privacy issues, but are available from the corresponding author on reasonable request.
