# Supplementary material for: Validation and refinement of a biomarker panel for frailty assessment and prediction of muscle weakness in older adults
Source: NPJ Aging. 2026 Sep 17;12(1):127. doi: 10.1038/s41514-026-00488-1 (PMC13586229; doi:10.1038/s41514-026-00488-1)
Supplement: Supplementary file 1 [file 41514_2026_488_MOESM1_ESM.pdf]

(a)

| Rank 1 | CV1 | CV2 | CV3 | CV4 | CV5 |
|---|---|---|---|---|---|
| Age | 1.030 [0.936-1.134] | 0.979 [0.891-1.076] | 1.001 [0.901-1.114] | 1.013 [0.924-1.111] | 1.012 [0.921-1.112] |
| Sex | 0.140 [0.025-0.785] | 0.273 [0.052-1.443] | 0.045 [0.006-0.365] | 0.185 [0.034-1.021] | 0.420 [0.077-2.285] |
| BMI | 1.701 [1.274-2.273] | 1.540 [1.202-1.973] | 2.092 [1.464-2.988] | 1.645 [1.245-2.175] | 1.420 [1.092-1.846] |
| SMI | 0.071 [0.018-0.288] | 0.114 [0.036-0.364] | 0.042 [0.009-0.202] | 0.105 [0.030-0.373] | 0.137 [0.042-0.445] |
| Apelin | 1.031 [1.010-1.053] | 1.022 [1.003-1.041] | 1.029 [1.006-1.052] | 1.028 [1.008-1.049] | 1.022 [1.003-1.042] |
| GDF15 | 3.759 [1.405-10.053] | 5.067 [1.855-13.845] | 10.838 [2.879-40.802] | 3.510 [1.391-8.857] | 6.599 [2.059-21.149] |
| CXCL10 | 1.004 [0.999-1.009] | 1.005 [0.999-1.010] | 1.003 [0.997-1.010] | 1.002 [0.998-1.007] | 1.004 [0.999-1.009] |

(b)

| Rank 2 | CV1 | CV2 | CV3 | CV4 | CV5 |
|---|---|---|---|---|---|
| Age | 1.060 [0.968-1.162] | 1.092 [0.991-1.203] | 0.995 [0.912-1.086] | 0.982 [0.895-1.079] | 1.017 [0.928-1.115] |
| Sex | 0.122 [0.022-0.693] | 0.122 [0.022-0.664] | 0.134 [0.028-0.637] | 0.117 [0.019-0.720] | 0.182 [0.033-1.005] |
| BMI | 1.696 [1.294-2.223] | 1.884 [1.365-2.601] | 1.630 [1.258-2.112] | 1.688 [1.270-2.245] | 1.649 [1.240-2.193] |
| SMI | 0.076 [0.021-0.277] | 0.047 [0.011-0.202] | 0.092 [0.028-0.307] | 0.087 [0.023-0.329] | 0.082 [0.023-0.296] |
| Apelin | 1.025 [1.006-1.045] | 1.037 [1.015-1.059] | 1.026 [1.007-1.046] | 1.031 [1.010-1.051] | 1.023 [1.005-1.042] |
| GDF15 | 6.444 [2.196-18.909] | 4.952 [1.855-13.220] | 4.995 [1.922-12.986] | 6.917 [2.284-20.948] | 5.804 [1.636-20.593] |

(c)

| Rank 3 | CV1 | CV2 | CV3 | CV4 | CV5 |
|---|---|---|---|---|---|
| Age | 0.999 [0.916-1.090] | 1.037 [0.948-1.134] | 1.019 [0.929-1.116] | 1.014 [0.923-1.114] | 1.053 [0.959-1.156] |
| Sex | 0.135 [0.028-0.656] | 0.195 [0.035-1.100] | 0.064 [0.010-0.420] | 0.285 [0.055-1.478] | 0.160 [0.028-0.916] |
| BMI | 1.666 [1.280-2.168] | 1.600 [1.220-2.097] | 1.725 [1.245-2.390] | 1.689 [1.271-2.245] | 1.692 [1.254-2.284] |
| SMI | 0.084 [0.024-0.291] | 0.094 [0.026-0.344] | 0.058 [0.013-0.267] | 0.094 [0.027-0.336] | 0.081 [0.020-0.319] |
| Apelin | 1.027 [1.006-1.047] | 1.035 [1.014-1.055] | 1.019 [9.998E-01-1.039] | 1.025 [1.006-1.045] | 1.029 [1.009-1.050] |
| GDF15 | 4.752 [1.734-13.022] | 4.657 [1.461-14.852] | 5.433 [1.866-15.820] | 4.205 [1.410-12.535] | 5.783 [1.868-17.900] |
| FGF21 | 1.001 [0.997-1.005] | 1.002 [0.998-1.005] | 1.002 [0.998-1.006] | 1.003 [0.999-1.007] | 1.001 [0.997-1.005] |

(d)

| Rank 4 | CV1 | CV2 | CV3 | CV4 | CV5 |
|---|---|---|---|---|---|
| Age | 0.995 [0.906-1.092] | 1.038 [0.948-1.138] | 1.037 [0.943-1.141] | 1.027 [0.941-1.120] | 1.029 [0.938-1.128] |
| Sex | 0.139 [0.025-0.780] | 0.262 [0.045-1.509] | 0.082 [0.013-0.523] | 0.192 [0.033-1.116] | 0.173 [0.032-0.936] |
| BMI | 1.574 [1.192-2.079] | 1.698 [1.268-2.274] | 1.813 [1.352-2.431] | 1.665 [1.230-2.252] | 1.597 [1.235-2.065] |
| SMI | 0.086 [0.024-0.306] | 0.061 [0.015-0.252] | 0.067 [0.018-0.255] | 0.097 [0.024-0.389] | 0.115 [0.034-0.388] |
| Apelin | 1.025 [1.006-1.044] | 1.032 [1.011-1.052] | 1.030 [1.008-1.052] | 1.023 [1.003-1.044] | 1.026 [1.006-1.046] |
| GDF15 | 6.474 [2.191-19.130] | 6.054 [2.005-18.275] | 6.853 [2.001-23.469] | 3.897 [1.430-10.619] | 4.737 [1.708-13.134] |
| TNFα | 1.063 [0.869-1.302] | 1.048 [0.847-1.297] | 1.022 [0.815-1.281] | 1.076 [0.870-1.331] | 1.074 [0.869-1.327] |

(e)

| Rank 5 | CV1 | CV2 | CV3 | CV4 | CV5 |
|---|---|---|---|---|---|
| Age | 0.990 [0.898-1.090] | 1.033 [0.937-1.139] | 0.950 [0.861-1.048] | 1.008 [0.922-1.103] | 1.051 [0.952-1.160] |
| Sex | 0.168 [0.029-0.978] | 0.360 [0.053-2.450] | 0.117 [0.021-0.662] | 0.243 [0.047-1.264] | 0.168 [0.029-0.978] |
| BMI | 1.660 [1.243-2.216] | 1.523 [1.160-2.001] | 1.607 [1.194-2.162] | 1.563 [1.192-2.049] | 1.748 [1.303-2.345] |
| SMI | 0.103 [0.028-0.385] | 0.137 [0.038-0.494] | 0.083 [0.021-0.319] | 0.100 [0.028-0.356] | 0.072 [0.019-0.279] |
| Apelin | 1.031 [1.010-1.053] | 1.024 [1.005-1.043] | 1.027 [1.006-1.048] | 1.023 [1.004-1.042] | 1.024 [1.002-1.046] |
| GDF15 | 5.942 [1.821-19.391] | 5.926 [1.849-18.990] | 4.783 [1.683-13.595] | 3.243 [1.185-8.879] | 4.916 [1.691-14.291] |
| CXCL10 | 1.004 [0.999-1.009] | 1.002 [0.997-1.007] | 1.003 [0.997-1.009] | 1.003 [0.998-1.008] | 1.006 [0.999-1.013] |
| FGF21 | 1.001 [0.997-1.004] | 1.003 [0.999-1.008] | 1.001 [0.997-1.005] | 1.000 [0.996-1.004] | 1.002 [0.998-1.007] |

(f)

| Base | CV1 | CV2 | CV3 | CV4 | CV5 |
|---|---|---|---|---|---|
| Age | 1.089 [1.019-1.165] | 1.056 [0.985-1.132] | 1.095 [1.024-1.170] | 1.110 [1.034-1.193] | 1.086 [1.012-1.167] |
| Sex | 1.092 [0.471-2.532] | 1.186 [0.531-2.650] | 0.882 [0.384-2.026] | 1.562 [0.670-3.642] | 1.507 [0.683-3.326] |
| BMI | 1.013 [0.922-1.112] | 1.034 [0.942-1.134] | 1.055 [0.962-1.156] | 1.094 [0.983-1.217] | 1.051 [0.950-1.163] |

**Supplementary Figure. 1. Odds ratio of 5-fold cross validation (CV) of top 5 (a-e) and base (f) models.**

Square brackets represent 95% confidence intervals. A white background indicates statistical significance ($P < 0.05$), whereas a gray background indicates no statistical significance.

**Supplementary Table 1. Linear regression analysis of blood test data**

| Item | Robust† | Pre-frail† | Frail† | *n* | β | *P* | Corrected *P* |
|---|---|---|---|---|---|---|---|
| MCHC (%) | 33.88 ± 1.12 | 33.06 ± 0.79 | 33.34 ± 1.11 | 167 | -0.20 | 2.21E-03 | 0.10 |
| 25-Hydroxyvitamin D (ng/mL) | 20.22 ± 6.26 | 16.12 ± 4.59 | 16.64 ± 6.10 | 167 | -3.45E-02 | 3.08E-03 | 0.14 |
| Red cell distribution width (%) | 13.02 ± 0.83 | 14.17 ± 1.70 | 13.69 ± 1.41 | 167 | 0.15 | 3.73E-03 | 0.17 |
| Mean corpuscular hemoglobin (%) | 31.37 ± 1.55 | 30.46 ± 1.84 | 30.62 ± 2.20 | 167 | -0.10 | 6.45E-03 | 0.30 |
| Free thyroxine 3 (pg/mL) | 2.64 ± 0.37 | 2.49 ± 0.31 | 2.47 ± 0.31 | 165 | -0.57 | 6.51E-03 | 0.30 |
| Albumin to globulin ratio | 1.52 ± 0.24 | 1.35 ± 0.26 | 1.39 ± 0.23 | 166 | -0.77 | 7.90E-03 | 0.36 |
| Cholinesterase (U/L) | 327.80 ± 62.03 | 296.48 ± 77.84 | 296.85 ± 65.01 | 167 | -2.99E-03 | 8.31E-03 | 0.38 |
| Lymphocytes (%) | 31.87 ± 8.42 | 29.66 ± 7.00 | 28.58 ± 6.91 | 166 | -2.21E-02 | 2.06E-02 | 0.95 |
| Neutrophils (%) | 58.08 ± 8.92 | 59.68 ± 7.57 | 61.39 ± 8.33 | 166 | 1.77E-02 | 3.32E-02 | 1.00 |
| TRACP-5b (mU/dL) | 412.62 ± 157.29 | 414.04 ± 160.84 | 481.91 ± 221.36 | 166 | 7.22E-04 | 4.74E-02 | 1.00 |
| C-reactive protein (mg/dL) | 0.09 ± 0.20 | 0.15 ± 0.25 | 0.18 ± 0.32 | 167 | 0.51 | 4.88E-02 | 1.00 |
| Bilirubin (mg/dL) | 0.70 ± 0.25 | 0.70 ± 0.41 | 0.59 ± 0.23 | 167 | -0.51 | 5.13E-02 | 1.00 |
| Hemoglobin (g/dL) | 13.19 ± 1.11 | 12.28 ± 1.41 | 12.70 ± 1.45 | 167 | -0.10 | 7.35E-02 | 1.00 |
| Calcium (mg/dL) | 9.33 ± 0.37 | 9.31 ± 0.36 | 9.44 ± 0.46 | 168 | 0.30 | 7.92E-02 | 1.00 |
| Undercarboxylated osteocalcin (ng/mL) | 4.43 ± 2.60 | 4.64 ± 3.30 | 5.97 ± 6.63 | 165 | 2.31E-02 | 0.10 | 1.00 |
| Platelet distribution width (%) | 10.75 ± 1.21 | 11.12 ± 2.09 | 11.14 ± 1.62 | 167 | 7.33E-02 | 0.11 | 1.00 |
| Mean platelet volume (fL) | 9.79 ± 0.69 | 10.00 ± 1.12 | 10.01 ± 0.81 | 167 | 0.14 | 0.11 | 1.00 |
| Total protein (g/dL) | 7.15 ± 0.43 | 7.17 ± 0.50 | 7.26 ± 0.49 | 166 | 0.24 | 0.12 | 1.00 |
| Mean corpuscular volume (fL) | 92.58 ± 3.70 | 92.25 ± 5.03 | 91.79 ± 5.29 | 167 | -2.18E-02 | 0.15 | 1.00 |
| Parathyroid hormone, intact (pg/mL) | 50.80 ± 18.11 | 46.00 ± 17.23 | 64.53 ± 79.29 | 167 | 1.64E-03 | 0.19 | 1.00 |
| Potassium (mEq/L) | 4.19 ± 0.33 | 4.08 ± 0.35 | 4.11 ± 0.39 | 168 | -0.25 | 0.19 | 1.00 |
| Albumin (g/dL) | 4.28 ± 0.25 | 4.07 ± 0.30 | 4.18 ± 0.34 | 167 | -0.29 | 0.21 | 1.00 |
| Chloride (mEq/L) | 104.72 ± 2.38 | 105.24 ± 2.82 | 104.20 ± 3.34 | 168 | -2.85E-02 | 0.24 | 1.00 |
| Free thyroxine 4 (ng/dL) | 1.03 ± 0.12 | 1.06 ± 0.12 | 1.05 ± 0.14 | 165 | 0.65 | 0.24 | 1.00 |
| Sodium (mEql/L) | 141.46 ± 2.29 | 141.24 ± 2.68 | 140.96 ± 2.18 | 168 | -3.53E-02 | 0.26 | 1.00 |
| Creatinine (mg/dL) | 0.75 ± 0.21 | 0.68 ± 0.15 | 0.84 ± 0.56 | 159 | 0.20 | 0.27 | 1.00 |
| Gamma-glutamyl transpeptidase (U/L) | 26.93 ± 26.30 | 25.72 ± 18.91 | 30.73 ± 31.60 | 168 | 2.70E-03 | 0.29 | 1.00 |
| Monocytes (%) | 7.09 ± 2.10 | 7.56 ± 2.39 | 6.77 ± 3.00 | 166 | -2.93E-02 | 0.31 | 1.00 |
| Eosinophils (%) | 2.34 ± 1.59 | 2.52 ± 1.43 | 2.70 ± 2.08 | 166 | 3.83E-02 | 0.34 | 1.00 |
| Hematocrit (%) | 38.92 ± 3.09 | 37.16 ± 3.75 | 38.06 ± 3.96 | 167 | -1.85E-02 | 0.37 | 1.00 |
| NTx (nmol BCE/L) | 15.35 ± 3.02 | 15.50 ± 4.49 | 16.08 ± 8.14 | 165 | 9.68E-03 | 0.40 | 1.00 |
| Inorganic phosphorus (mg/dL) | 3.63 ± 0.44 | 3.60 ± 0.50 | 3.59 ± 0.58 | 167 | -0.10 | 0.45 | 1.00 |

| Item | Robust† | Pre-frail† | Frail† | *n* | β | *P* | Corrected *P* |
|---|---|---|---|---|---|---|---|
| Platelets (×104/μL) | 21.32 ± 3.81 | 24.33 ± 6.61 | 21.75 ± 5.12 | 167 | 1.10E-02 | 0.48 | 1.00 |
| Basophils (%) | 0.62 ± 0.30 | 0.56 ± 0.32 | 0.57 ± 0.36 | 166 | -0.14 | 0.53 | 1.00 |
| Aspartate aminotransferase (U/L) | 22.56 ± 6.06 | 22.92 ± 7.36 | 23.70 ± 9.20 | 167 | 4.93E-03 | 0.59 | 1.00 |
| High-density lipoprotein (mg/dL) | 59.56 ± 14.16 | 57.32 ± 12.26 | 58.27 ± 14.03 | 156 | -3.01E-03 | 0.60 | 1.00 |
| Total cholesterol (mg/dL) | 195.57 ± 35.82 | 193.36 ± 36.32 | 196.56 ± 31.52 | 167 | 8.59E-04 | 0.69 | 1.00 |
| White blood cells (×102/μL) | 53.71 ± 10.09 | 61.60 ± 15.75 | 54.98 ± 13.48 | 167 | 1.80E-03 | 0.75 | 1.00 |
| Urea nitrogen (mg/dL) | 18.43 ± 5.25 | 17.17 ± 4.87 | 19.29 ± 13.13 | 159 | 2.17E-03 | 0.77 | 1.00 |
| Creatine kinase (U/L) | 108.07 ± 52.32 | 125.64 ± 114.80 | 109.33 ± 62.99 | 168 | -1.84E-04 | 0.86 | 1.00 |
| eGFR (mL/min/1.73 m2) | 66.55 ± 14.40 | 71.17 ± 14.68 | 64.46 ± 18.70 | 159 | -7.74E-04 | 0.87 | 1.00 |
| Amylase (U/L) | 88.02 ± 30.56 | 81.80 ± 25.64 | 86.09 ± 36.68 | 168 | -3.00E-04 | 0.89 | 1.00 |
| Red blood cells (×104/μL) | 421.28 ± 39.56 | 404.32 ± 47.80 | 415.62 ± 45.88 | 167 | -1.66E-04 | 0.92 | 1.00 |
| Alanine aminotransferase (U/L) | 19.25 ± 8.81 | 19.96 ± 8.94 | 19.14 ± 8.53 | 167 | -1.43E-04 | 0.99 | 1.00 |

† Mean ± 1 SD, Corrected *P*; Bonferroni-corrected *P*

**Supplementary Table 2. Linear regression analysis of clinical data**

| Clinical data | Robust† | Pre-frail† | Frail† | *n* | β | *P* | Corrected *P* |
|---|---|---|---|---|---|---|---|
| **SMI [DEXA] (kg/m$^2$)** | 6.62 ± 0.91 | 6.31 ± 0.90 | 6.24 ± 1.12 | 168 | -0.6 | 5.32E-06 | **3.72E-05** |
| Heart rate (bpm) | 72.57 ± 10.38 | 86.76 ± 15.41 | 77.49 ± 11.80 | 168 | 1.30E-02 | 1.95E-02 | 0.14 |
| Years of education | 12.07 ± 2.44 | 11.44 ± 2.27 | 10.76 ± 2.94 | 168 | -6.13E-02 | 2.60E-02 | 0.18 |
| YAM‡ of lumbar spine (%) | 95.08 ± 24.52 | 98.64 ± 21.66 | 90.63 ± 22.78 | 168 | -4.83E-03 | 0.19 | 1.00 |
| Diastolic blood pressure (mmHg) | 79.87 ± 11.95 | 77.40 ± 12.09 | 77.07 ± 13.18 | 168 | -6.91E-03 | 0.22 | 1.00 |
| YAM‡ of femoral neck (%) | 80.77 ± 13.72 | 85.22 ± 12.07 | 77.71 ± 15.58 | 166 | -4.99E-03 | 0.38 | 1.00 |
| Systolic blood pressure (mmHg) | 147.44 ± 19.88 | 140.20 ± 18.27 | 146.44 ± 23.58 | 168 | -1.69E-03 | 0.61 | 1.00 |

† Mean ± 1 SD, ‡ Young Adult Mean, Corrected *P*; Bonferroni-corrected *P*, Bold letters; Corrected $P < 0.05$

**Supplementary Table 3. Characterization by J-CHS criteria**

| Category | Total | Shrinking | | Weakness | | Exhaustion | | Slowness | | Low activity | |
|---|---|---|---|---|---|---|---|---|---|---|---|
| | | 1 | 0 | 1 | 0 | 1 | 0 | 1 | 0 | 1 | 0 |
| Number of samples | 168 | 30 | 138 | 55 | 113 | 64 | 104 | 98 | 70 | 73 | 95 |
| Age (years)† | 77.3 ± 6.1 | 78.4 ± 5.3 | 77.0 ± 6.3 | 80.0 ± 5.9 | 76.0 ± 5.8 | 78.8 ± 5.5 | 76.3 ± 6.3 | 78.0 ± 6.4 | 76.3 ± 5.6 | 77.0 ± 6.6 | 77.5 ± 5.7 |
| Sex ratio (male:female) | 1:2.17 | 1:2.00 | 1:2.21 | 1:2.24 | 1:2.14 | 1:2.37 | 1:2.06 | 1:2.63 | 1:1.69 | 1:2.04 | 1:2.28 |
| BMI (kg/m$^2$)† | 24.49 ± 4.08 | 23.03 ± 4.44 | 24.8 ± 3.94 | 23.73 ± 4.55 | 24.85 ± 3.80 | 24.28 ± 4.55 | 24.62 ± 3.78 | 24.90 ± 4.76 | 23.9 ± 2.80 | 25.39 ± 4.99 | 23.79 ± 3.06 |
| Clinical data† | | | | | | | | | | | |
| SMI [DEXA] (kg/m$^2$) | 6.39 ± 1.03 | 6.08 ± 0.98 | 6.46 ± 1.03 | 6.00 ± 1.08 | 6.58 ± 0.95 | 6.24 ± 1.04 | 6.48 ± 1.02 | 6.29 ± 1.08 | 6.53 ± 0.94 | 6.39 ± 1.14 | 6.39 ± 0.94 |
| ELISA data† | | | | | | | | | | | |
| GDF15 (ng/mL) | 1.55 ± 0.93 | 2.32 ± 1.60 | 1.38 ± 0.60 | 2.02 ± 1.26 | 1.32 ± 0.61 | 1.83 ± 1.19 | 1.38 ± 0.68 | 1.61 ± 0.75 | 1.46 ± 1.14 | 1.63 ± 0.86 | 1.49 ± 0.98 |
| Apelin (ng/mL) | 129.07 ± 30.46 | 147.80 ± 22.88 | 125.00 ± 30.44 | 140.36 ± 28.48 | 123.57 ± 29.99 | 138.78 ± 29.67 | 123.09 ± 29.52 | 132.88 ± 31.65 | 123.73 ± 28.08 | 130.61 ± 29.98 | 127.89 ± 30.93 |
| CXCL10 (pg/mL) | 158.23 ± 146.74 | 201.89 ± 182.66 | 148.73 ± 136.67 | 228.26 ± 198.66 | 124.14 ± 97.46 | 190.06 ± 148.11 | 138.63 ± 143.10 | 182.67 ± 164.39 | 124.00 ± 109.95 | 177.26 ± 161.04 | 143.60 ± 133.76 |
| TNFα (pg/mL) | 3.26 ± 3.51 | 5.43 ± 4.44 | 2.79 ± 3.09 | 4.43 ± 4.09 | 2.69 ± 3.04 | 3.65 ± 2.89 | 3.02 ± 3.83 | 3.59 ± 3.62 | 2.81 ± 3.31 | 3.85 ± 3.72 | 2.81 ± 3.28 |
| FGF21 (pg/mL) | 276.67 ± 307.31 | 467.94 ± 611.22 | 235.09 ± 162.13 | 364.29 ± 473.59 | 234.02 ± 164.53 | 351.50 ± 446.84 | 230.62 ± 159.28 | 280.35 ± 199.18 | 271.51 ± 415.63 | 295.16 ± 209.75 | 262.46 ± 365.53 |

† Mean ± 1 SD

**Supplementary Table 4. Linear regression analysis of age-related factors**

| ELISA data | Robust† | Pre-frail† | Frail† | *n* | β | *P* | Corrected *P* |
|---|---|---|---|---|---|---|---|
| **GDF15 (ng/mL)** | 1.18 ± 0.42 | 1.39 ± 0.64 | 1.87 ± 1.15 | 168 | 0.32 | 3.11E-05 | **3.11E-04** |
| **Apelin (ng/mL)** | 121.41 ± 27.77 | 107.53 ± 30.20 | 141.33 ± 27.06 | 168 | 8.58E-03 | 2.31E-04 | **2.31E-03** |
| **CXCL10 (pg/mL)** | 110.58 ± 84.61 | 118.66 ± 138.76 | 205.73 ± 170.03 | 168 | 1.79E-03 | 5.70E-04 | **5.70E-03** |
| **TNFα (pg/mL)** | 2.27 ± 2.43 | 2.32 ± 4.75 | 4.28 ± 3.50 | 168 | 7.02E-02 | 1.01E-03 | **1.01E-02** |
| **FGF21 (pg/mL)** | 189.90 ± 100.36 | 247.14 ± 173.78 | 350.22 ± 408.51 | 168 | 6.51E-04 | 4.27E-03 | **4.27E-02** |
| Progranulin (ng/mL) | 155.80 ± 32.12 | 172.08 ± 33.85 | 172.85 ± 39.04 | 168 | 4.66E-03 | 1.74E-02 | 0.17 |
| Adiponectin (µg/mL) | 9.30 ± 4.83 | 12.34 ± 6.98 | 11.61 ± 7.40 | 168 | 2.53E-02 | 2.53E-02 | 0.25 |
| CXCL9 (pg/mL) | 160.62 ± 195.83 | 260.57 ± 269.69 | 241.96 ± 236.39 | 168 | 5.95E-04 | 5.34E-02 | 0.53 |
| BDNF (ng/mL) | 11.39 ± 4.10 | 15.43 ± 3.75 | 12.59 ± 4.70 | 168 | 2.58E-02 | 0.10 | 1.00 |
| eNAMPT (ng/mL) | 0.93 ± 0.72 | 1.24 ± 0.54 | 1.01 ± 1.09 | 168 | 5.50E-02 | 0.48 | 1.00 |

† Mean ± 1 SD, Corrected *P*; Bonferroni-corrected *P*, Bold letters; Corrected *P* < 0.05

**Supplementary Table 5. Standardization of RNA quality control and RNA-seq data**

| ID | RIN | Input reads | Unique map reads | % Unique map reads | Multi-map reads | % Multi-map reads |
|---|---|---|---|---|---|---|
| Robust 1 | 7.3 | 25,272,783 | 20,677,361 | 81.82 | 3,260,587 | 12.9 |
| Robust 2 | 7.3 | 26,052,454 | 21,744,323 | 83.46 | 2,490,335 | 9.56 |
| Robust 3 | 7.7 | 24,612,964 | 21,799,757 | 88.57 | 2,123,757 | 8.63 |
| Robust 4 | 6.9 | 39,422,691 | 33,731,284 | 85.56 | 4,710,328 | 11.95 |
| Robust 5 | 7.3 | 29,925,604 | 25,504,558 | 85.23 | 3,540,701 | 11.83 |
| Robust 6 | 6.8 | 30,409,060 | 25,929,588 | 85.27 | 3,673,449 | 12.08 |
| Robust 7 | 6.9 | 26,740,929 | 23,067,679 | 86.26 | 2,915,311 | 10.9 |
| Robust 8 | 7.9 | 30,160,225 | 25,886,868 | 85.83 | 3,422,710 | 11.35 |
| Robust 9 | 7.9 | 29,516,026 | 24,819,280 | 84.09 | 3,493,190 | 11.83 |
| Robust 10 | 8.2 | 29,935,556 | 26,289,712 | 87.82 | 2,851,083 | 9.52 |
| Robust 11 | 8.5 | 36,406,877 | 31,624,226 | 86.86 | 3,909,181 | 10.74 |
| Robust 12 | 7.8 | 29,227,318 | 24,987,270 | 85.49 | 3,200,079 | 10.95 |
| Robust 13 | 7.7 | 22,725,853 | 18,594,694 | 81.82 | 3,218,532 | 14.16 |
| Robust 14 | 8 | 28,694,942 | 24,145,231 | 84.14 | 3,511,425 | 12.24 |
| Robust 15 | 8.5 | 19,093,730 | 16,228,395 | 84.99 | 2,331,416 | 12.21 |
| Robust 16 | 8.1 | 20,688,428 | 16,824,178 | 81.32 | 2,802,523 | 13.55 |
| Robust 17 | 7.2 | 37,308,513 | 33,402,646 | 89.53 | 3,291,606 | 8.82 |
| Robust 18 | 8.1 | 27,414,468 | 22,939,334 | 83.68 | 3,970,692 | 14.48 |
| Robust 19 | 7.7 | 31,077,527 | 26,927,509 | 86.65 | 2,884,850 | 9.28 |
| Robust 20 | 7.7 | 26,666,755 | 22,330,164 | 83.74 | 3,327,079 | 12.48 |
| Robust 21 | 6.4 | 26,078,395 | 23,147,510 | 88.76 | 2,203,198 | 8.45 |
| Robust 22 | 8.4 | 31,279,922 | 26,573,622 | 84.95 | 3,640,298 | 11.64 |
| Robust 23 | 8.1 | 30,774,733 | 25,093,383 | 81.54 | 4,652,339 | 15.12 |
| Robust 24 | 7.6 | 35,498,436 | 30,061,866 | 84.69 | 4,237,424 | 11.94 |
| Robust 25 | 6.3 | 34,757,575 | 29,713,386 | 85.49 | 3,939,525 | 11.33 |
| Robust 26 | 6.9 | 19,415,412 | 16,032,032 | 82.57 | 1,971,162 | 10.15 |
| Robust 27 | 6.3 | 31,147,799 | 25,280,541 | 81.16 | 4,635,615 | 14.88 |
| Robust 28 | 7.3 | 25,371,494 | 19,098,600 | 75.28 | 3,116,491 | 12.28 |
| Robust 29 | 7.6 | 59,992,047 | 49,457,416 | 82.44 | 7,721,530 | 12.87 |
| Robust 30 | 7.1 | 14,881,161 | 12,349,793 | 82.99 | 1,336,532 | 8.98 |
| Robust 31 | 7.6 | 26,853,477 | 21,443,128 | 79.85 | 3,503,922 | 13.05 |
| Robust 32 | 6.7 | 23,114,076 | 18,895,602 | 81.75 | 2,562,679 | 11.09 |
| Robust 33 | 8.2 | 27,528,547 | 22,898,131 | 83.18 | 3,075,377 | 11.17 |
| Robust 34 | 7.4 | 35,273,486 | 28,061,448 | 79.55 | 4,930,322 | 13.98 |
| Robust 35 | 7.9 | 26,325,956 | 21,783,040 | 82.74 | 2,988,429 | 11.35 |
| Robust 36 | 7.2 | 34,298,969 | 28,382,064 | 82.75 | 3,620,103 | 10.55 |
| Robust 37 | 7.2 | 20,076,444 | 17,492,693 | 87.13 | 2,069,108 | 10.31 |
| Robust 38 | 8.1 | 36,584,381 | 32,100,891 | 87.74 | 3,407,289 | 9.31 |
| Robust 39 | 8.4 | 57,143,524 | 48,892,387 | 85.56 | 6,469,925 | 11.32 |
| Robust 40 | 6.9 | 39,327,966 | 32,397,951 | 82.38 | 5,757,350 | 14.64 |
| Robust 41 | 7.7 | 41,042,130 | 36,450,781 | 88.81 | 3,507,615 | 8.55 |
| Robust 42 | 7.9 | 27,406,357 | 21,083,791 | 76.93 | 4,368,843 | 15.94 |
| Robust 43 | 7.2 | 27,336,506 | 23,011,340 | 84.18 | 3,597,956 | 13.16 |
| Robust 44 | 6.4 | 26,044,803 | 23,504,015 | 90.24 | 1,083,596 | 4.16 |
| Robust 45 | 7.7 | 30,341,821 | 26,797,992 | 88.32 | 1,805,137 | 5.95 |
| Robust 46 | 6.7 | 42,560,919 | 35,410,792 | 83.2 | 4,572,490 | 10.74 |
| Robust 47 | 7.3 | 26,476,387 | 22,129,504 | 83.58 | 2,712,946 | 10.25 |
| Robust 48 | 6.8 | 22,668,576 | 18,929,247 | 83.5 | 2,421,017 | 10.68 |
| Robust 49 | 7.2 | 25,864,928 | 22,730,805 | 87.88 | 1,641,352 | 6.35 |
| Robust 50 | 6.9 | 22,420,102 | 19,934,640 | 88.91 | 1,325,607 | 5.91 |

| ID | RIN | Input reads | Unique map reads | % Unique map reads | Multi-map reads | % Multi-map reads |
|---|---|---|---|---|---|---|
| Robust 51 | 7.3 | 44,227,920 | 39,165,179 | 88.55 | 2,322,039 | 5.25 |
| Robust 52 | 7.7 | 29,796,975 | 26,473,065 | 88.84 | 1,591,631 | 5.34 |
| Robust 53 | 7.2 | 27,202,783 | 23,391,735 | 85.99 | 2,757,557 | 10.14 |
| Robust 54 | 7.2 | 20,973,245 | 17,309,932 | 82.53 | 2,327,006 | 11.1 |
| Robust 55 | 6.7 | 61,677,624 | 51,202,161 | 83.02 | 7,047,718 | 11.43 |
| Robust 56 | 7 | 24,121,195 | 20,360,463 | 84.41 | 2,267,236 | 9.4 |
| Robust 57 | 6.5 | 22,228,008 | 19,108,044 | 85.96 | 1,684,760 | 7.58 |
| Robust 58 | 6.3 | 31,442,410 | 27,645,269 | 87.92 | 1,965,426 | 6.25 |
| Robust 59 | 6.7 | 24,533,337 | 21,729,013 | 88.57 | 1,258,999 | 5.13 |
| Robust 60 | 6.6 | 18,258,048 | 16,151,358 | 88.46 | 764,606 | 4.19 |
| Robust 61 | 6.5 | 28,951,158 | 23,690,344 | 81.83 | 3,212,307 | 11.1 |
| PreFrail 1 | 6.4 | 30,444,754 | 24,529,846 | 80.57 | 3,250,704 | 10.68 |
| PreFrail 2 | 8.2 | 44,572,569 | 33,346,643 | 74.81 | 8,343,282 | 18.72 |
| PreFrail 3 | 7.4 | 35,901,906 | 31,755,753 | 88.45 | 3,087,541 | 8.6 |
| PreFrail 4 | 6 | 21,120,020 | 18,640,970 | 88.26 | 1,328,591 | 6.29 |
| PreFrail 5 | 6.8 | 25,936,068 | 23,211,112 | 89.49 | 949,996 | 3.66 |
| PreFrail 6 | 8.1 | 26,715,437 | 23,672,831 | 88.61 | 1,476,988 | 5.53 |
| PreFrail 7 | 7 | 18,590,817 | 15,783,869 | 84.9 | 1,757,851 | 9.46 |
| PreFrail 8 | 6.5 | 23,610,136 | 18,825,504 | 79.73 | 3,221,163 | 13.64 |
| PreFrail 9 | 6.4 | 28,457,220 | 23,159,565 | 81.38 | 2,989,645 | 10.51 |
| PreFrail 10 | 6.5 | 31,940,023 | 25,296,669 | 79.2 | 4,993,696 | 15.63 |
| PreFrail 11 | 7.9 | 19,091,880 | 14,589,717 | 76.42 | 1,791,112 | 9.38 |
| PreFrail 12 | 6.9 | 23,131,878 | 18,558,985 | 80.23 | 3,212,250 | 13.89 |
| PreFrail 13 | 7.2 | 22,724,003 | 18,368,960 | 80.84 | 2,956,192 | 13.01 |
| PreFrail 14 | 7.2 | 30,766,760 | 26,063,097 | 84.71 | 3,726,596 | 12.11 |
| PreFrail 15 | 8.4 | 36,256,771 | 30,420,605 | 83.9 | 3,898,070 | 10.75 |
| PreFrail 16 | 8.1 | 22,896,535 | 18,138,833 | 79.22 | 4,099,654 | 17.91 |
| PreFrail 17 | 8 | 17,406,258 | 14,012,851 | 80.5 | 2,242,536 | 12.88 |
| PreFrail 18 | 7.4 | 22,792,397 | 20,361,361 | 89.33 | 1,130,085 | 4.96 |
| PreFrail 19 | 6.9 | 26,533,500 | 23,562,741 | 88.8 | 1,701,146 | 6.41 |
| PreFrail 20 | 7.4 | 26,501,633 | 22,266,557 | 84.02 | 2,402,052 | 9.06 |
| PreFrail 21 | 6.7 | 23,704,943 | 18,112,580 | 76.41 | 3,623,884 | 15.29 |
| PreFrail 22 | 6.4 | 17,353,735 | 14,838,139 | 85.5 | 1,281,440 | 7.38 |
| PreFrail 23 | 7.7 | 33,225,241 | 29,416,425 | 88.54 | 2,300,521 | 6.92 |
| PreFrail 24 | 7.5 | 22,049,080 | 18,957,398 | 85.98 | 1,673,747 | 7.59 |
| PreFrail 25 | 6.2 | 32,345,564 | 27,183,439 | 84.04 | 3,345,165 | 10.34 |
| Frail 1 | 6.3 | 24,224,695 | 20,010,408 | 82.6 | 2,582,782 | 10.66 |
| Frail 2 | 6.2 | 20,208,334 | 16,739,230 | 82.83 | 2,123,540 | 10.51 |
| Frail 3 | 6.4 | 30,945,136 | 25,707,055 | 83.07 | 3,189,893 | 10.31 |
| Frail 4 | 6.4 | 28,733,957 | 23,013,919 | 80.09 | 3,608,201 | 12.56 |
| Frail 5 | 6.9 | 32,334,474 | 25,031,572 | 77.41 | 5,024,281 | 15.54 |
| Frail 6 | 6.5 | 21,792,092 | 18,105,790 | 83.08 | 2,144,376 | 9.84 |
| Frail 7 | 6.1 | 31,288,894 | 26,150,970 | 83.58 | 2,991,881 | 9.56 |
| Frail 8 | 7.1 | 24,434,091 | 18,202,934 | 74.5 | 2,722,227 | 11.14 |
| Frail 9 | 7.5 | 25,114,542 | 20,137,468 | 80.18 | 3,552,981 | 14.15 |
| Frail 10 | 7.7 | 22,105,703 | 18,260,147 | 82.6 | 2,423,376 | 10.96 |
| Frail 11 | 7.9 | 27,435,320 | 21,740,100 | 79.24 | 4,051,717 | 14.77 |
| Frail 12 | 7.6 | 21,255,769 | 17,218,425 | 81.01 | 2,337,563 | 11 |
| Frail 13 | 7.5 | 25,105,320 | 20,035,078 | 79.8 | 3,506,220 | 13.97 |
| Frail 14 | 7 | 26,145,537 | 21,819,091 | 83.45 | 2,495,642 | 9.55 |
| Frail 15 | 7.7 | 33,919,625 | 26,034,355 | 76.75 | 5,542,086 | 16.34 |

| ID | RIN | Input reads | Unique map reads | % Unique map reads | Multi-map reads | % Multi-map reads |
|---|---|---|---|---|---|---|
| Frail 16 | 8.7 | 18,739,505 | 14,033,368 | 74.89 | 3,443,865 | 18.38 |
| Frail 17 | 6.5 | 24,433,545 | 19,115,874 | 78.24 | 3,508,743 | 14.36 |
| Frail 18 | 7.9 | 28,408,766 | 22,539,697 | 79.34 | 3,932,099 | 13.84 |
| Frail 19 | 7.3 | 21,633,817 | 16,291,076 | 75.3 | 3,740,614 | 17.29 |
| Frail 20 | 6.8 | 18,643,800 | 13,924,676 | 74.69 | 3,515,784 | 18.86 |
| Frail 21 | 7.6 | 14,762,190 | 10,924,472 | 74 | 2,149,919 | 14.56 |
| Frail 22 | 6.9 | 34,150,787 | 28,421,532 | 83.22 | 3,664,306 | 10.73 |
| Frail 23 | 7.7 | 27,756,342 | 23,642,689 | 85.18 | 2,510,022 | 9.04 |
| Frail 24 | 7.4 | 29,003,982 | 23,683,716 | 81.66 | 3,283,012 | 11.32 |
| Frail 25 | 8.2 | 27,678,701 | 22,160,231 | 80.06 | 3,590,614 | 12.97 |
| Frail 26 | 7.3 | 31,604,449 | 25,059,024 | 79.29 | 4,921,292 | 15.57 |
| Frail 27 | 8.6 | 26,556,394 | 22,077,740 | 83.14 | 3,003,515 | 11.31 |
| Frail 28 | 8.2 | 34,563,043 | 28,404,861 | 82.18 | 4,163,870 | 12.05 |
| Frail 29 | 7.5 | 29,649,125 | 24,960,661 | 84.19 | 2,830,982 | 9.55 |
| Frail 30 | 7.8 | 30,380,154 | 24,403,122 | 80.33 | 4,297,549 | 14.15 |
| Frail 31 | 7.9 | 27,851,942 | 22,793,060 | 81.84 | 3,082,302 | 11.07 |
| Frail 32 | 7.4 | 30,525,085 | 22,875,426 | 74.94 | 5,793,072 | 18.98 |
| Frail 33 | 6.2 | 23,034,612 | 19,296,008 | 83.77 | 3,176,919 | 13.79 |
| Frail 34 | 8.5 | 26,127,824 | 18,920,004 | 72.41 | 5,265,616 | 20.15 |
| Frail 35 | 6.8 | 37,684,347 | 31,608,209 | 83.88 | 4,476,832 | 11.88 |
| Frail 36 | 7.7 | 33,955,255 | 26,506,824 | 78.06 | 5,917,988 | 17.43 |
| Frail 37 | 7 | 28,091,943 | 21,566,128 | 76.77 | 4,981,108 | 17.73 |
| Frail 38 | 7.5 | 25,120,664 | 21,493,668 | 85.56 | 2,980,158 | 11.86 |
| Frail 39 | 8 | 19,768,348 | 16,100,051 | 81.44 | 2,547,050 | 12.88 |
| Frail 40 | 6.1 | 32,417,365 | 28,847,856 | 88.99 | 2,907,979 | 8.97 |
| Frail 41 | 6.8 | 31,268,767 | 26,440,349 | 84.56 | 3,914,248 | 12.52 |
| Frail 42 | 6.2 | 34,167,171 | 29,220,775 | 85.52 | 4,101,702 | 12 |
| Frail 43 | 7.2 | 22,817,698 | 18,476,841 | 80.98 | 3,735,006 | 16.37 |
| Frail 44 | 6.1 | 40,314,330 | 35,265,265 | 87.48 | 4,118,538 | 10.22 |
| Frail 45 | 7 | 26,822,625 | 23,020,632 | 85.83 | 2,944,537 | 10.98 |
| Frail 46 | 7.5 | 33,518,339 | 29,171,734 | 87.03 | 3,421,069 | 10.21 |
| Frail 47 | 7.2 | 20,827,190 | 17,621,750 | 84.61 | 2,699,868 | 12.96 |
| Frail 48 | 7.4 | 48,811,270 | 41,900,533 | 85.84 | 5,839,337 | 11.96 |
| Frail 49 | 8.1 | 36,994,364 | 31,105,526 | 84.08 | 4,952,400 | 13.39 |
| Frail 50 | 8.1 | 42,526,089 | 37,539,538 | 88.27 | 3,966,860 | 9.33 |
| Frail 51 | 7.4 | 29,521,503 | 25,388,372 | 86 | 3,360,374 | 11.38 |
| Frail 52 | 7.4 | 30,714,113 | 25,813,151 | 84.04 | 3,669,751 | 11.95 |
| Frail 53 | 7.5 | 28,015,431 | 23,889,326 | 85.27 | 2,953,865 | 10.54 |
| Frail 54 | 7.8 | 34,086,642 | 29,126,154 | 85.45 | 3,973,341 | 11.66 |
| Frail 55 | 7.8 | 45,207,102 | 37,676,982 | 83.34 | 5,581,027 | 12.35 |
| Frail 56 | 7.6 | 26,954,943 | 23,151,678 | 85.89 | 3,004,699 | 11.15 |
| Frail 57 | 7.5 | 36,373,275 | 29,693,245 | 81.63 | 5,607,957 | 15.42 |
| Frail 58 | 7.3 | 22,405,117 | 16,326,800 | 72.87 | 4,142,343 | 18.49 |
| Frail 59 | 7.4 | 31,198,903 | 26,228,475 | 84.07 | 3,199,567 | 10.26 |
| Frail 60 | 8 | 22,288,159 | 18,410,664 | 82.6 | 2,498,518 | 11.21 |
| Frail 61 | 7.4 | 38,789,688 | 31,973,360 | 82.43 | 4,508,165 | 11.62 |
| Frail 62 | 7.8 | 26,925,435 | 23,444,162 | 87.07 | 1,960,735 | 7.28 |
| Frail 63 | 6 | 16,010,107 | 13,412,913 | 83.78 | 1,462,829 | 9.14 |
| Frail 64 | 7.1 | 21,597,630 | 17,579,406 | 81.4 | 2,500,172 | 11.58 |
| Frail 65 | 6.7 | 20,615,243 | 17,283,193 | 83.84 | 1,908,795 | 9.26 |
| Frail 66 | 8.3 | 14,989,954 | 12,152,952 | 81.07 | 1,662,828 | 11.09 |

| ID | RIN | Input reads | Unique map reads | % Unique map reads | Multi-map reads | % Multi-map reads |
|---|---|---|---|---|---|---|
| Frail 67 | 7 | 25,142,750 | 20,334,621 | 80.88 | 2,870,259 | 11.42 |
| Frail 68 | 7.4 | 16,455,874 | 12,058,455 | 73.28 | 2,002,320 | 12.17 |
| Frail 69 | 7.8 | 23,868,982 | 19,275,466 | 80.76 | 2,847,054 | 11.93 |
| Frail 70 | 8 | 25,782,699 | 19,988,765 | 77.53 | 3,790,512 | 14.7 |
| Frail 71 | 7.8 | 18,321,954 | 14,685,473 | 80.15 | 2,417,094 | 13.19 |
| Frail 72 | 7.1 | 20,337,524 | 16,442,415 | 80.85 | 2,630,750 | 12.94 |
| Frail 73 | 7.3 | 23,346,035 | 18,992,610 | 81.35 | 2,629,675 | 11.26 |
| Frail 74 | 6.9 | 21,107,169 | 16,313,377 | 77.29 | 3,107,748 | 14.72 |
| Frail 75 | 7.9 | 16,601,829 | 11,675,327 | 70.33 | 2,892,305 | 17.42 |
| Frail 76 | 7.4 | 34,496,270 | 29,342,678 | 85.06 | 2,999,827 | 8.7 |
| Frail 77 | 7.1 | 31,081,782 | 27,277,714 | 87.76 | 2,928,286 | 9.42 |
| Frail 78 | 7.4 | 34,530,221 | 28,936,013 | 83.8 | 4,621,827 | 13.38 |
| Frail 79 | 7.4 | 33,827,691 | 27,912,868 | 82.51 | 4,957,493 | 14.66 |
| Frail 80 | 7 | 28,318,605 | 22,983,945 | 81.16 | 3,099,433 | 10.94 |
| Frail 81 | 7 | 22,040,396 | 16,665,577 | 75.61 | 3,592,755 | 16.3 |
| Frail 82 | 7.8 | 18,930,731 | 15,698,025 | 82.92 | 2,009,602 | 10.62 |

## Supplementary Table 6. Genes associated with frailty

| Ensembl Gene ID | β | Corrected *P* |
|---|---|---|
| ENSG00000196268.7 | -0.106 | 1.626E-05 |
| ENSG00000257621.3 | -0.048 | 9.069E-05 |
| ENSG00000176018.8 | -0.189 | 1.960E-04 |
| ENSG00000121931.11 | -0.319 | 2.643E-04 |
| ENSG00000120837.3 | -0.278 | 4.024E-04 |
| ENSG00000182141.5 | -0.154 | 5.410E-04 |
| ENSG00000105866.9 | -0.230 | 5.572E-04 |
| ENSG00000013441.11 | -0.016 | 6.823E-04 |
| ENSG00000168876.4 | -0.104 | 6.966E-04 |
| ENSG00000167232.9 | -0.052 | 7.396E-04 |
| ENSG00000166012.10 | -0.031 | 9.486E-04 |
| ENSG00000111615.8 | -0.087 | 1.029E-03 |
| ENSG00000100483.9 | -0.241 | 1.125E-03 |
| ENSG00000100578.10 | -0.189 | 1.628E-03 |
| ENSG00000113971.14 | -0.201 | 1.936E-03 |
| ENSG00000174796.8 | -0.273 | 2.073E-03 |
| ENSG00000071243.11 | -0.160 | 2.313E-03 |
| ENSG00000132424.10 | -0.022 | 2.453E-03 |
| ENSG00000000419.8 | -0.132 | 2.763E-03 |
| ENSG00000145725.15 | -0.044 | 2.938E-03 |
| ENSG00000142166.8 | -0.078 | 3.116E-03 |
| ENSG00000160229.7 | -0.224 | 3.173E-03 |
| ENSG00000189266.7 | -0.044 | 3.187E-03 |
| ENSG00000047346.8 | -0.061 | 3.393E-03 |
| ENSG00000140307.6 | -0.112 | 3.421E-03 |
| ENSG00000138041.11 | -0.070 | 3.568E-03 |
| ENSG00000164548.6 | -0.058 | 3.592E-03 |
| ENSG00000120686.7 | -0.070 | 3.713E-03 |
| ENSG00000144034.10 | -0.181 | 3.924E-03 |
| ENSG00000268027.1 | -0.072 | 4.015E-03 |
| ENSG00000118418.10 | -0.076 | 4.163E-03 |
| ENSG00000127993.10 | -0.348 | 4.253E-03 |
| ENSG00000188243.8 | -0.025 | 4.308E-03 |
| ENSG00000198642.5 | -0.193 | 4.416E-03 |
| ENSG00000168216.6 | -0.081 | 4.477E-03 |
| ENSG00000185946.11 | -0.106 | 4.685E-03 |
| ENSG00000164180.9 | -0.192 | 4.720E-03 |
| ENSG00000111011.13 | -0.052 | 4.734E-03 |
| ENSG00000105879.7 | -0.170 | 5.402E-03 |
| ENSG00000163328.9 | -0.219 | 5.426E-03 |
| ENSG00000205981.2 | -0.157 | 5.523E-03 |
| ENSG00000168283.9 | -0.106 | 5.542E-03 |
| ENSG00000145782.8 | -0.069 | 5.696E-03 |
| ENSG00000141446.6 | -0.185 | 5.981E-03 |
| ENSG00000148943.7 | -0.215 | 6.068E-03 |
| ENSG00000115839.13 | -0.141 | 6.165E-03 |
| ENSG00000262944.1 | -0.093 | 6.244E-03 |
| ENSG00000196850.4 | -0.145 | 6.450E-03 |
| ENSG00000134077.11 | -0.230 | 6.560E-03 |
| ENSG00000075303.8 | -0.165 | 6.566E-03 |

| | | |
|---|---|---|
| ENSG00000158290.12 | -0.097 | 6.762E-03 |
| ENSG00000196199.9 | -0.121 | 6.801E-03 |
| ENSG00000176390.10 | -0.055 | 6.809E-03 |
| ENSG00000087338.4 | -0.215 | 7.110E-03 |
| ENSG00000177853.10 | -0.107 | 7.261E-03 |
| ENSG00000155957.12 | -0.037 | 7.265E-03 |
| ENSG00000145439.7 | -0.216 | 7.269E-03 |
| ENSG00000104343.15 | -0.096 | 7.314E-03 |
| ENSG00000116266.6 | -0.041 | 7.503E-03 |
| ENSG00000179387.5 | -0.162 | 7.833E-03 |
| ENSG00000107938.13 | -0.150 | 7.993E-03 |
| ENSG00000139726.6 | -0.221 | 8.063E-03 |
| ENSG00000144401.10 | -0.171 | 8.087E-03 |
| ENSG00000143742.8 | -0.031 | 8.144E-03 |
| ENSG00000144048.6 | -0.176 | 8.330E-03 |
| ENSG00000122417.11 | -0.062 | 8.712E-03 |
| ENSG00000106460.14 | -0.164 | 8.891E-03 |
| ENSG00000034533.7 | -0.322 | 9.037E-03 |
| ENSG00000096746.13 | -0.091 | 9.381E-03 |
| ENSG00000152219.4 | -0.137 | 9.428E-03 |
| ENSG00000236287.3 | -0.107 | 9.774E-03 |
| ENSG00000111711.5 | -0.152 | 9.949E-03 |
| ENSG00000139746.11 | -0.085 | 1.005E-02 |
| ENSG00000253738.1 | -0.233 | 1.020E-02 |
| ENSG00000107679.10 | -0.160 | 1.033E-02 |
| ENSG00000179456.9 | -0.140 | 1.034E-02 |
| ENSG00000133703.7 | -0.129 | 1.035E-02 |
| ENSG00000119820.6 | -0.070 | 1.053E-02 |
| ENSG00000177683.9 | -0.123 | 1.076E-02 |
| ENSG00000179454.9 | -0.212 | 1.133E-02 |
| ENSG00000141646.9 | -0.051 | 1.135E-02 |
| ENSG00000176102.7 | -0.242 | 1.160E-02 |
| ENSG00000163867.12 | -0.169 | 1.185E-02 |
| ENSG00000162613.12 | -0.028 | 1.185E-02 |
| ENSG00000151665.8 | -0.141 | 1.189E-02 |
| ENSG00000116954.7 | -0.232 | 1.191E-02 |
| ENSG00000198862.9 | -0.124 | 1.198E-02 |
| ENSG00000147601.9 | -0.214 | 1.199E-02 |
| ENSG00000145740.14 | -0.160 | 1.204E-02 |
| ENSG00000061987.10 | -0.046 | 1.222E-02 |
| ENSG00000129292.16 | -0.030 | 1.276E-02 |
| ENSG00000172086.7 | -0.132 | 1.290E-02 |
| ENSG00000107560.6 | -0.159 | 1.313E-02 |
| ENSG00000163001.7 | -0.194 | 1.330E-02 |
| ENSG00000170852.6 | -0.072 | 1.331E-02 |
| ENSG00000219545.5 | -0.246 | 1.337E-02 |
| ENSG00000134049.3 | -0.196 | 1.351E-02 |
| ENSG00000122033.10 | -0.137 | 1.353E-02 |
| ENSG00000134186.7 | -0.081 | 1.382E-02 |
| ENSG00000134294.9 | -0.029 | 1.383E-02 |
| ENSG00000100632.6 | -0.057 | 1.416E-02 |
| ENSG00000086189.5 | -0.114 | 1.433E-02 |

| | | |
|---|---|---|
| ENSG00000113593.7 | -0.047 | 1.462E-02 |
| ENSG00000047932.9 | -0.209 | 1.480E-02 |
| ENSG00000198743.5 | -0.239 | 1.485E-02 |
| ENSG00000100567.8 | -0.052 | 1.507E-02 |
| ENSG00000169193.7 | -0.104 | 1.564E-02 |
| ENSG00000260822.1 | -0.191 | 1.573E-02 |
| ENSG00000214367.3 | -0.223 | 1.594E-02 |
| ENSG00000086589.7 | -0.156 | 1.605E-02 |
| ENSG00000213020.4 | -0.223 | 1.611E-02 |
| ENSG00000152518.5 | -0.060 | 1.623E-02 |
| ENSG00000143633.8 | -0.250 | 1.633E-02 |
| ENSG00000150776.13 | -0.253 | 1.638E-02 |
| ENSG00000146282.13 | -0.161 | 1.641E-02 |
| ENSG00000172466.11 | -0.092 | 1.655E-02 |
| ENSG00000196247.7 | -0.184 | 1.661E-02 |
| ENSG00000218739.5 | -0.273 | 1.677E-02 |
| ENSG00000110367.7 | -0.020 | 1.708E-02 |
| ENSG00000246263.2 | -0.153 | 1.710E-02 |
| ENSG00000084073.4 | -0.178 | 1.738E-02 |
| ENSG00000065135.7 | -0.068 | 1.763E-02 |
| ENSG00000148634.11 | -0.042 | 1.770E-02 |
| ENSG00000108854.11 | -0.164 | 1.788E-02 |
| ENSG00000121749.11 | -0.082 | 1.789E-02 |
| ENSG00000014123.9 | -0.219 | 1.797E-02 |
| ENSG00000131013.3 | -0.152 | 1.809E-02 |
| ENSG00000010244.12 | -0.028 | 1.816E-02 |
| ENSG00000114416.13 | -0.046 | 1.820E-02 |
| ENSG00000171566.7 | -0.096 | 1.829E-02 |
| ENSG00000163608.10 | -0.114 | 1.862E-02 |
| ENSG00000136451.4 | -0.139 | 1.896E-02 |
| ENSG00000165169.6 | -0.217 | 1.918E-02 |
| ENSG00000173890.12 | -0.138 | 1.919E-02 |
| ENSG00000198961.5 | -0.042 | 1.920E-02 |
| ENSG00000149532.11 | -0.155 | 1.925E-02 |
| ENSG00000144381.12 | -0.071 | 1.934E-02 |
| ENSG00000125304.8 | -0.055 | 1.968E-02 |
| ENSG00000162601.5 | -0.097 | 2.020E-02 |
| ENSG00000188647.8 | -0.058 | 2.024E-02 |
| ENSG00000113240.8 | -0.055 | 2.025E-02 |
| ENSG00000095951.12 | -0.148 | 2.084E-02 |
| ENSG00000133983.10 | -0.184 | 2.097E-02 |
| ENSG00000129128.8 | -0.043 | 2.106E-02 |
| ENSG00000164754.8 | -0.041 | 2.145E-02 |
| ENSG00000163320.6 | -0.056 | 2.146E-02 |
| ENSG00000088035.11 | -0.139 | 2.171E-02 |
| ENSG00000197045.8 | -0.113 | 2.212E-02 |
| ENSG00000171109.14 | -0.076 | 2.236E-02 |
| ENSG00000139218.13 | -0.021 | 2.246E-02 |
| ENSG00000164941.9 | -0.056 | 2.303E-02 |
| ENSG00000054219.9 | -0.078 | 2.371E-02 |
| ENSG00000171497.4 | -0.202 | 2.375E-02 |
| ENSG00000135999.7 | -0.095 | 2.406E-02 |

| | | |
|---|---|---|
| ENSG00000133872.9 | -0.018 | 2.453E-02 |
| ENSG00000163602.9 | -0.175 | 2.462E-02 |
| ENSG00000135341.13 | -0.126 | 2.475E-02 |
| ENSG00000119396.6 | -0.108 | 2.477E-02 |
| ENSG00000005812.6 | -0.060 | 2.545E-02 |
| ENSG00000082258.8 | -0.079 | 2.550E-02 |
| ENSG00000147251.11 | -0.043 | 2.590E-02 |
| ENSG00000129007.10 | -0.229 | 2.607E-02 |
| ENSG00000148153.9 | -0.218 | 2.633E-02 |
| ENSG00000110172.7 | -0.109 | 2.640E-02 |
| ENSG00000164985.10 | -0.039 | 2.664E-02 |
| ENSG00000180182.6 | -0.180 | 2.674E-02 |
| ENSG00000069943.5 | -0.046 | 2.737E-02 |
| ENSG00000122481.12 | -0.130 | 2.743E-02 |
| ENSG00000135336.10 | -0.213 | 2.749E-02 |
| ENSG00000135185.7 | -0.058 | 2.781E-02 |
| ENSG00000111897.6 | -0.027 | 2.786E-02 |
| ENSG00000135974.5 | -0.327 | 2.790E-02 |
| ENSG00000156313.8 | -0.103 | 2.851E-02 |
| ENSG00000102974.10 | -0.283 | 2.874E-02 |
| ENSG00000228655.2 | -0.091 | 2.996E-02 |
| ENSG00000142867.8 | -0.280 | 2.998E-02 |
| ENSG00000162961.9 | -0.152 | 3.056E-02 |
| ENSG00000139372.10 | -0.191 | 3.087E-02 |
| ENSG00000144357.12 | -0.074 | 3.125E-02 |
| ENSG00000119707.9 | -0.056 | 3.147E-02 |
| ENSG00000234511.4 | -0.107 | 3.155E-02 |
| ENSG00000183291.11 | -0.032 | 3.178E-02 |
| ENSG00000116191.13 | -0.054 | 3.183E-02 |
| ENSG00000149311.13 | -0.011 | 3.186E-02 |
| ENSG00000163714.13 | -0.044 | 3.237E-02 |
| ENSG00000271533.1 | -0.122 | 3.242E-02 |
| ENSG00000137040.8 | -0.172 | 3.257E-02 |
| ENSG00000115084.8 | -0.075 | 3.302E-02 |
| ENSG00000166479.5 | -0.071 | 3.326E-02 |
| ENSG00000119541.5 | -0.075 | 3.331E-02 |
| ENSG00000164631.14 | -0.182 | 3.350E-02 |
| ENSG00000183891.5 | -0.141 | 3.376E-02 |
| ENSG00000164031.12 | -0.068 | 3.388E-02 |
| ENSG00000145780.6 | -0.260 | 3.391E-02 |
| ENSG00000145241.6 | -0.082 | 3.406E-02 |
| ENSG00000091140.8 | -0.087 | 3.459E-02 |
| ENSG00000133226.12 | -0.079 | 3.553E-02 |
| ENSG00000058673.11 | -0.046 | 3.587E-02 |
| ENSG00000196470.7 | -0.193 | 3.589E-02 |
| ENSG00000171988.13 | -0.014 | 3.615E-02 |
| ENSG00000029363.11 | -0.026 | 3.635E-02 |
| ENSG00000182004.8 | -0.065 | 3.641E-02 |
| ENSG00000115935.12 | -0.023 | 3.663E-02 |
| ENSG00000079134.7 | -0.071 | 3.664E-02 |
| ENSG00000134717.13 | -0.132 | 3.692E-02 |
| ENSG00000090054.9 | -0.119 | 3.695E-02 |

| | | |
|---|---|---|
| ENSG00000149231.7 | -0.069 | 3.696E-02 |
| ENSG00000141367.7 | -0.039 | 3.739E-02 |
| ENSG00000196705.4 | -0.182 | 3.807E-02 |
| ENSG00000081237.14 | -0.003 | 3.859E-02 |
| ENSG00000138398.11 | -0.070 | 3.860E-02 |
| ENSG00000168522.8 | -0.071 | 3.869E-02 |
| ENSG00000112282.13 | -0.094 | 3.871E-02 |
| ENSG00000108064.6 | -0.147 | 3.881E-02 |
| ENSG00000119285.6 | -0.189 | 3.924E-02 |
| ENSG00000143207.15 | -0.020 | 4.018E-02 |
| ENSG00000127914.12 | -0.055 | 4.031E-02 |
| ENSG00000146802.8 | -0.186 | 4.053E-02 |
| ENSG00000173230.11 | -0.099 | 4.094E-02 |
| ENSG00000107854.5 | -0.124 | 4.148E-02 |
| ENSG00000167562.7 | -0.217 | 4.182E-02 |
| ENSG00000080345.13 | -0.095 | 4.346E-02 |
| ENSG00000115540.10 | -0.125 | 4.355E-02 |
| ENSG00000138768.10 | -0.094 | 4.360E-02 |
| ENSG00000116752.5 | -0.175 | 4.365E-02 |
| ENSG00000138381.5 | -0.095 | 4.367E-02 |
| ENSG00000047410.9 | -0.035 | 4.386E-02 |
| ENSG00000153339.9 | -0.067 | 4.397E-02 |
| ENSG00000081665.9 | -0.098 | 4.404E-02 |
| ENSG00000036257.8 | -0.044 | 4.406E-02 |
| ENSG00000114742.9 | -0.079 | 4.442E-02 |
| ENSG00000066777.4 | -0.075 | 4.455E-02 |
| ENSG00000188321.9 | -0.174 | 4.473E-02 |
| ENSG00000105708.8 | -0.313 | 4.501E-02 |
| ENSG00000166913.8 | -0.027 | 4.579E-02 |
| ENSG00000138468.11 | -0.065 | 4.602E-02 |
| ENSG00000162928.8 | -0.327 | 4.603E-02 |
| ENSG00000121274.8 | -0.327 | 4.649E-02 |
| ENSG00000159593.10 | -0.104 | 4.677E-02 |
| ENSG00000174579.3 | -0.092 | 4.699E-02 |
| ENSG00000146457.10 | -0.045 | 4.755E-02 |
| ENSG00000140006.7 | -0.238 | 4.798E-02 |
| ENSG00000137876.5 | -0.060 | 4.833E-02 |
| ENSG00000186298.7 | -0.038 | 4.876E-02 |
| ENSG00000211456.6 | -0.062 | 4.884E-02 |
| ENSG00000164331.5 | -0.091 | 4.924E-02 |
| ENSG00000092199.13 | -0.014 | 4.926E-02 |
| ENSG00000115020.12 | -0.114 | 4.931E-02 |
| ENSG00000129071.5 | -0.091 | 4.969E-02 |
| ENSG00000039319.12 | -0.049 | 4.990E-02 |
| ENSG00000110321.11 | -0.021 | 4.999E-02 |

Corrected *P*; Bonferroni-corrected *P*

**Supplementary Table 7. Correlations between RNA-seq gene expression levels (TPM) and ELISA-measured protein concentrations**

| ELISA | Gene symbol | TPM† | *r* | *P* | Corrected *P* |
|---|---|---|---|---|---|
| **CXCL10** | ***CXCL10*** | 0.60 ± 0.54 | 0.33 | **1.45E-05** | **1.31E-04** |
| **GDF15** | ***GDF15*** | 0.22 ± 0.16 | 0.26 | **7.90E-04** | **7.11E-03** |
| **eNAMPT** | ***NAMPT*** | 505.75 ± 234.35 | 0.21 | **5.53E-03** | **4.98E-02** |
| Progranulin | *GRN* | 18.50 ± 8.79 | 0.15 | 5.17E-02 | 0.46 |
| TNFα | *TNF* | 0.50 ± 0.21 | 0.08 | 0.31 | 1.00 |
| CXCL9 | *CXCL9* | 4.33E-02 ± 4.03E-02 | 0.07 | 0.35 | 1.00 |
| Apelin | *APLN* | 2.98E-04 ± 2.03E-03 | -0.04 | 0.61 | 1.00 |
| BDNF | *BDNF* | 3.60E-02 ± 2.45E-02 | 0.04 | 0.64 | 1.00 |
| Adiponectin | *ADIPOQ* | 3.57E-04 ± 1.86E-03 | -0.01 | 0.94 | 1.00 |
| FGF21 | *FGF21* | 0.00 ± 0.00 | N.A. | N.A. | N.A. |

† Mean ± 1 SD, *r*; Pearson correlation coefficient, Bold letters; *P* < 0.05; N.A.; Not applicable due to zero variance, Corrected *P*; Bonferroni-corrected *P*

**Supplementary Table 8. Characterization of prospective data of robust subjects**

| Category | Total | Shrinking | | Weakness | | Exhaustion | | Slowness | | Low activity | |
|---|---|---|---|---|---|---|---|---|---|---|---|
| | | 1 | 0 | 1 | 0 | 1 | 0 | 1 | 0 | 1 | 0 |
| Number of samples | 37 | 4 | 33 | 7 | 30 | 1 | 36 | 1 | 36 | 2 | 35 |
| Event rate (%) | 35.1 | 10.8 | - | 18.9 | - | 2.7 | - | 2.7 | - | 5.4 | - |
| Age (years)† | 77.7 ± 5.2 | 76.3 ± 5.7 | 77.8 ± 5.2 | 80.3 ± 4.2 | 77.0 ± 5.2 | 77 | 77.7 ± 5.2 | 90 | 77.3 ± 4.8 | 74.0 ± 11.3 | 77.9 ± 4.9 |
| Sex ratio (male:female) | 1:1.85 | 1:3.00 | 1:1.75 | 1:6.00 | 1:1.50 | 1 female | 1:1.77 | 1 female | 1:1.77 | 1:1.00 | 1:1.92 |
| BMI (kg/m$^2$)† | 24.19 ± 2.65 | 23.73 ± 2.05 | 24.25 ± 2.73 | 24.29 ± 1.98 | 24.17 ± 2.81 | 24.7 | 24.18 ± 2.68 | 21.1 | 24.28 ± 2.63 | 26.40 ± 2.40 | 24.07 ± 2.63 |
| Follow-up duration (days)† | 607.7 ± 482.1 | 385.8 ± 23.5 | 634.6 ± 504.5 | 605.6 ± 368.3 | 608.2 ± 510.4 | 364.0 | 614.5 ± 487.2 | 1819.0 | 574.1 ± 442.7 | 1122.5 ± 1006.2 | 578.3 ± 447.0 |
| Clinical data† | | | | | | | | | | | |
| SMI [DEXA] (kg/m$^2$) | 6.65 ± 0.84 | 6.26 ± 0.83 | 6.70 ± 0.84 | 6.23 ± 0.59 | 6.75 ± 0.87 | 6.18 | 6.66 ± 0.85 | 6.20 | 6.66 ± 0.85 | 7.11 ± 0.13 | 6.62 ± 0.86 |
| ELISA data† | | | | | | | | | | | |
| GDF15 (ng/mL) | 1.17 ± 0.43 | 1.34 ± 0.27 | 1.15 ± 0.45 | 1.34 ± 0.46 | 1.13 ± 0.43 | 1.61 | 1.16 ± 0.43 | 0.94 | 1.18 ± 0.44 | 0.83 ± 0.43 | 1.19 ± 0.43 |
| Apelin (ng/mL) | 125.73 ± 28.01 | 127.98 ± 13.33 | 125.45 ± 29.42 | 104.61 ± 30.17 | 130.65 ± 25.55 | 126.24 | 125.71 ± 28.41 | 142.96 | 125.25 ± 28.25 | 93.59 ± 49.32 | 127.56 ± 26.36 |

† Mean ± 1 SD
